# SOX4 facilitates brown fat development and maintenance through EBF2-mediated thermogenic gene program in mice

**DOI:** 10.1038/s41418-024-01397-0

**Published:** 2024-10-15

**Authors:** Shuai Wang, Ting He, Ya Luo, Kexin Ren, Huanming Shen, Lingfeng Hou, Yixin Wei, Tong Fu, Wenlong Xie, Peng Wang, Jie Hu, Yu Zhu, Zhengrong Huang, Qiyuan Li, Weihua Li, Huiling Guo, Boan Li

**Affiliations:** 1 State Key Laboratory of Cellular Stress Biology, Innovation Center for Cell Signaling Network and Engineering Research Center of Molecular Diagnostics of The Ministry of Education, School of Life Sciences, Xiamen University, 361102 Xiamen, Fujian China; 2 Department of Cardiology, Xiamen Key Laboratory of Cardiac Electrophysiology, Xiamen Institute of Cardiovascular Diseases, The First Affiliated Hospital of Xiamen University, School of Medicine, Xiamen University, 361102 Xiamen, China; 3 National Institute for Data Science in Health and Medicine, School of Medicine, Xiamen University, 361102 Xiamen, Fujian China; 4 Shenzhen Institute of Advanced Technology, Chinese Academy of Science, 518055 Shenzhen, China

**Keywords:** Cell biology, Endocrine system and metabolic diseases

## Abstract

Brown adipose tissue (BAT) is critical for non-shivering thermogenesis making it a promising therapeutic strategy to combat obesity and metabolic disease. However, the regulatory mechanisms underlying brown fat formation remain incompletely understood. Here, we found SOX4 is required for BAT development and thermogenic program. Depletion of SOX4 in BAT progenitors (*Sox4-MKO*) or brown adipocytes (*Sox4-BKO*) resulted in whitened BAT and hypothermia upon acute cold exposure. The reduced thermogenic capacity of *Sox4-MKO* mice increases their susceptibility to diet-induced obesity. Conversely, overexpression of SOX4 in BAT enhances thermogenesis counteracting diet-induced obesity. Mechanistically, SOX4 activates the transcription of EBF2, which determines brown fat fate. Moreover, phosphorylation of SOX4 at S235 by PKA facilitates its nuclear translocation and EBF2 transcription. Further, SOX4 cooperates with EBF2 to activate transcriptional programs governing thermogenic gene expression. These results demonstrate that SOX4 serves as an upstream regulator of EBF2, providing valuable insights into BAT development and thermogenic function maintenance.

## Introduction

Obesity occurs when energy intake from food exceeds energy expenditure. Adipose tissues are important for regulating whole-body energy homeostasis [1]. White adipose tissue (WAT) consists mainly of white adipocytes that store excess energy in fat. Brown adipose tissue (BAT), on the other hand, utilizes lipids and glucose to generate heat, which is essential for maintaining body temperature and resisting cold-induced hypothermia in mammals [2–5]. In brown adipocytes, activation of uncoupling protein-1 (Ucp1) which is located in the inner membrane of mitochondria causes proton leakage resulting in energy dissipation as heat. Mice with enhanced thermogenic activity of BAT exhibited increased energy expenditure, resulting in resistance to weight gain [6]. Conversely, mice lacking BAT thermogenic function were more susceptible to developing obesity [7]. Intensive studies have elucidated that metabolically active brown fat is present in both infants and adults [8]. And the increased BAT activity of human is correlated with declining body weight [3, 9, 10]. Therefore, promoting BAT development and function might be a promising strategy to counteract obesity and related metabolic disorders [6, 11, 12].

Several transcriptional regulators have been shown to participate in BAT development and function. The previous lineage studies clarified that classic brown adipocytes derived from multipotent progenitor cells that express engrailed 1 (*En1*), paired box protein 3 (*Pax3*), *Pax*7, and myogenic factor 5 (*Myf5*) in the somatic mesoderm during embryogenesis [13]. During BAT development, these progenitors develop to preadipocytes and subsequently differentiate to brown adipocytes with a transcriptional cascade. Early B-cell factor 2 (EBF2) is an essential transcriptional regulator for brown fat cell fate. It could recruit peroxisome proliferator-activated receptor-γ (PPARγ), a key transcription factor during adipocyte differentiation, to its brown-selective binding sites to maintain BAT identity [14, 15]. Removing the inhibitory effect on EBF2 enhances the thermogenic potential of white adipocytes [16]. Recent studies have demonstrated that EBF2 enhances the transcriptional activity of an ERRα/PGC1α complex to promote expression of thermogenic genes and maintain core temperature in BAT [17]. These findings indicate EBF2 is crucial for brown adipocyte differentiation and thermogenic function. However, the transcriptional regulator of *Ebf2* remains unknown.

SRY-related High Mobility Group box transcription factor 4 (SOX4), a member of Sox C family, contains high-mobility group DNA-binding domain (HMG-Box domain) and C-terminal transactivation domain (TAD domain) [18]. It plays a key role in regulating cell stemness, cell differentiation, and development of nervous system, endocrine islet, and heart [19–21]. We found that SOX4 not only suppresses preadipocyte determination in WAT [22], but also acts as a coactivator of PPARγ to facilitate beige adipocyte formation during prolonged cold exposure [23]. Although the regulation of brown and beige fat differentiation involves an overlapping set of pan-adipogenic and BAT-specific transcription factors [13], the potential involvement of SOX4 in determining the fate of brown adipocytes remains unclear.

Here, we show that SOX4 is required for BAT development and thermogenic function in mice by promoting the transcription of *Ebf2*, which determines brown fat fate. This process could be facilitated by PKA activation. Our findings elucidate the regulatory role and mechanism of SOX4 in controlling thermogenic gene programs, and suggest that targeting SOX4 may provide a potential strategy for enhancing energy expenditure to combat obesity.

## Results

### SOX4 is required for BAT development and maintenance

**Fig. 1:**
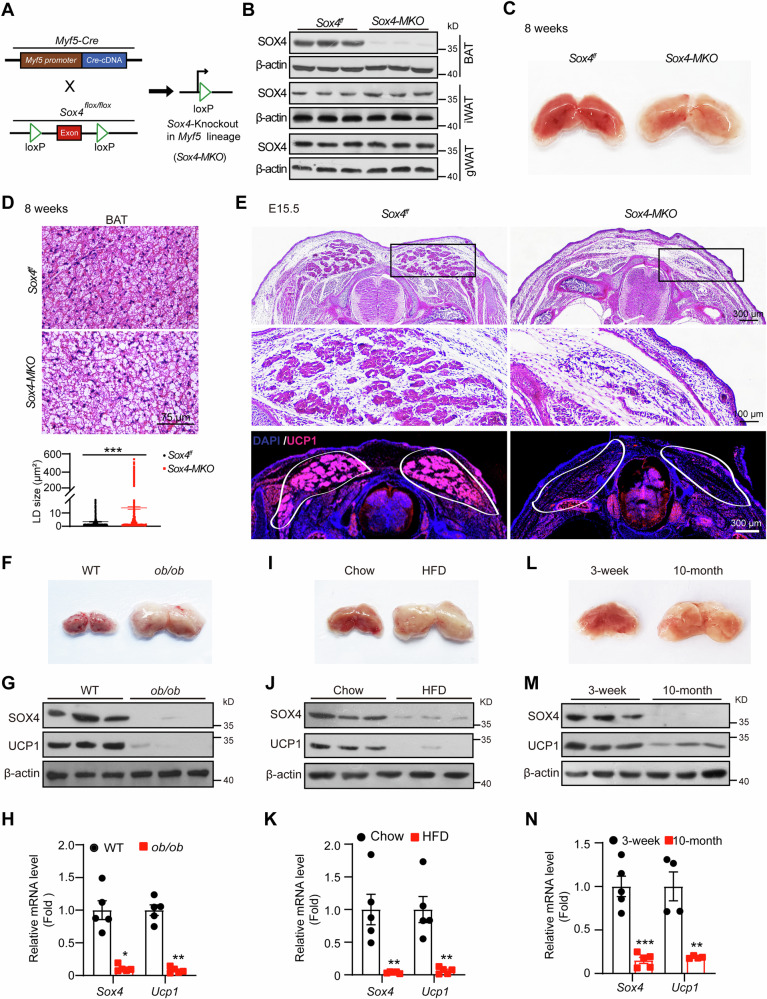
SOX4 is required for BAT development and maintenance. **A** The strategy of generating *Sox4-MKO* is achieved by intercrossing *Myf5-*Cre mice with *Sox4*^*f/f*^ mice. **B** The protein levels of SOX4 in BAT, iWAT and gWAT of *Sox4-MKO* mice and control male mice (8-week-old, n = 3). **C** Representative images of BAT isolated from 8-week-old *Sox4*^*f/f*^ *and Sox4-MKO* male mice. **D** Representative images of H&E staining of BAT (top panel, Scale bar, 75 μm) and the quantification of lipid droplets size from BAT (bottom panel) (***p < 0.001, data are presented as mean ± SEM, statistical analysis were determined by unpaired two-tailed Mann-Whitney test). **E** H&E staining and immunofluorescence (IF) analysis of representative sections from the interscapular regions of E15.5 *Sox4*^*f/f*^ and *Sox4-MKO* embryos. Scale bars are as indicated. **F**–**N** Analysis of BATs isolated from WT and *ob/ob* male mice (16-week-old, n = 5) (**F**–**H**), chow-diet-fed and HFD-fed male mice (16-week-old, n = 5) (**I**–**K**), 3-week-old and 10-month-old male mice (n = 5) (**L**–**N**). Representative images of BATs were shown in (**F**, **I**, **L**). Western blot analysis of SOX4 and UCP1 (**G**, **J**, **M**). qRT-PCR analysis of the mRNA levels of *Sox4* and *Ucp1* (**H**, **K**, **N**)*. 18S* was used as an invariant control. The mRNA levels in control mice were normalized to 1.0. Asterisks (*) denote the level of statistical significance. *p < 0.05, **p < 0.01, ***p < 0.001. Data are presented as mean ± SEM. Statistical analyses were determined by unpaired two-tailed Student’s *t*-test (**K**) and unpaired two-tailed Mann-Whitney test (**H**, **N**).

Our previous study demonstrated the requirement of SOX4 for beige adipocyte formation during prolonged cold exposure [23]. Here, we found SOX4 was mainly expressed in BAT and gonadal WAT (gWAT) (Fig. S1A, B). Notably, the mRNA levels of Sox4 in BAT stromal vascular fraction SVF cells (SVFs) were significantly higher than those in WAT SVFs (Fig. S1C). Following a 4-hour acute cold exposure, SOX4 mRNA levels exhibited a marked increase in both BAT tissues and BAT SVFs, while remaining unchanged in inguinal WAT (iWAT) and gWAT (Fig. S1D–F). Consequently, we sought to investigate the role of SOX4 in BAT, which is crucial for non-shivering thermogenesis. As BAT originates from *Myf5*^+^ precursor cells, we generated *Myf5-Cre Sox4*^*f/f*^ mice (referred to as *Sox4-MKO*) by crossing *Sox4*^*f/f*^ with *Myf5-Cre* mice (Fig. 1A). As shown in Fig. 1B, SOX4 expression was greatly diminished in BAT of *Sox4-MKO* mice. Additionally, the BATs from adult or newborn *Sox4-MKO* mice exhibited a whitened phenotype compared to that of wild type mice (Figs. 1C and  S1G). H&E staining further revealed enlarged lipid droplets in BATs of *Sox4-MKO* mice (Figs. 1D and  S1H). Since BAT develops during embryogenesis in murine models [6], we examined the development of BAT at E15.5 embryos from *Sox4-MKO* mice. As depicted in Fig. 1E, BAT depots in *Sox4-MKO* mice diminished compared to that of *Sox4*^*f/f*^ littermates and UCP1 expression was also obviously decreased. To further evaluate the role of SOX4 in BAT development, we generated BAT-specific *Sox4* knockout mice (*Sox4-BKO*) by crossing *Sox4*^*f/f*^ with *Ucp1-Cre* mice (Fig. S1I). SOX4 was specifically deleted in BAT but not in WAT (Fig. S1J). Similar to the observations made in *Sox4-MKO* mice, whitened BATs were observed in *Sox4-BKO* mice (Fig. S1K) characterized by enlarged lipid droplets (Fig. S1L). Notably, whitening of BAT is also observed in obese and older animals [24–26]. Accordingly, significant downregulation of SOX4 expression was detected in whitened BATs from *ob/ob* mice (Fig. 1F–H), high-fat diet-induced obese mice (Fig. 1I–K), and aged mice (Fig. 1L–N). Collectively, these findings suggested that SOX4 is essential for both the development and maintenance of BAT.

### SOX4 is required for thermogenesis of BAT

**Fig. 2:**
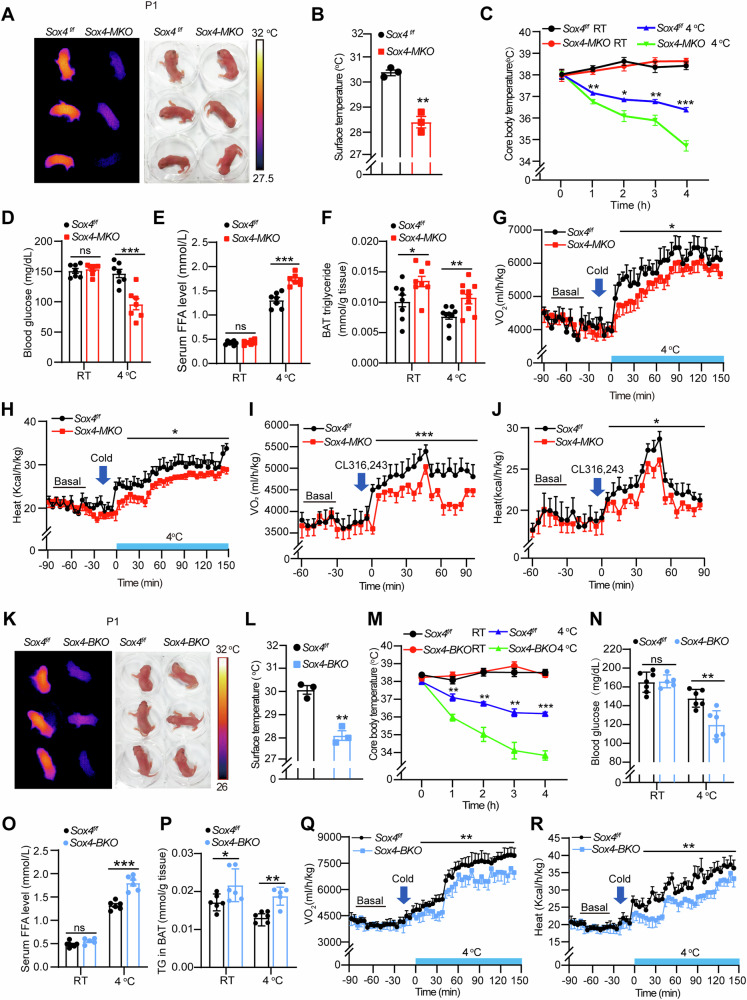
SOX4 is required for thermogenesis of BAT. **A**, **B** Infrared imaging of newborns (P1, male mice, n = 3) of *Sox4-MKO* and control littermates (**A**), and their skin temperatures from the infrared images were quantified and shown in (**B**). **C**–**F** Changes in core body temperature of *Sox4*^*f/f*^ *and Sox4-MKO* male mice (8-weeks-old, male) at room temperature (*Sox4*^*f/f*^, n = 7; *Sox4-MKO*, n = 8) or during acute cold exposure at 4 °C for 4 hours (*Sox4*^*f/f*^, n = 4; *Sox4-MKO*, n = 6) (**C**). After cold tolerance test (CTT), blood glucose was collected from tail vein and the levels of blood glucose was measured (n = 7) (**D**). The serum levels of free fatty acid (FFA) were measured (n = 7) (**E**). Triglyceride (TG) content in BAT tissue was measured at the end of the experiment ((RT: *Sox4*^*f/f*^, n = 8; *Sox4-MKO*, n = 8), (4 °C: *Sox4*^*f/f*^, n = 9; *Sox4-MKO*, n = 10)) (**F**). **G**, **H** *Sox4*^*f/f*^ *and Sox4-MKO* male mice (9-weeks-old, male) were switched from 22 °C to 4 °C, and the oxygen consumption (**G**) and heat production (**H**) were monitored by metabolic cage for 150 min (n = 6). **I**, **J** *Sox4*^*f/f*^ *and Sox4-MKO* male mice (8-weeks-old, male) were intraperitoneally injected with CL316,243 (1 mg/kg), and the oxygen consumption (**I**) and heat production (**J**) were monitored using metabolic cage (n = 6). **K**, **L** Infrared imaging of newborns (P1, male mice, n = 3) from *Sox4-BKO* and respective control mice (**K**), and their skin temperatures from the infrared images were quantified and shown in (**L**). **M**–**P** Changes in core body temperature changes of *Sox4-BKO* and control male mice (8-weeks old) at room temperature (*Sox4*^*f/f*^, n = 7; *Sox4-BKO*, n = 8) or 4 °C for 4 h (*Sox4*^*f/f*^, n = 4; *Sox4-BKO*, n = 6) (**M**). Blood glucose levels (**N**) and serum levels of FFA (**O**) were measured after the cold tolerance test ((RT: *Sox4*^*f/f*^, n = 7; *Sox4-BKO*, n = 5), (4 °C: *Sox4*^*f/f*^, n = 6; *Sox4-BKO*, n = 6)). TG content in BAT tissue was measured at the end of the experiment ((RT: *Sox4*^*f/f*^, n = 6; *Sox4-BKO*, n = 6), (4 °C: *Sox4*^*f/f*^, n = 6; *Sox4-BKO*, n = 5)) (**P**). **Q**, **R** *Sox4*^*f/f*^ *and Sox4-BKO* male mice (9-weeks-old) were switched from 22 °C to 4 °C, and the oxygen consumption (**Q**) and heat production (**R**) were monitored using metabolic cage for 150 min (n = 5). Asterisks (*) denote the level of statistical significance. ns, no significance, *p < 0.05, **p < 0.01, ***p < 0.001. Data are presented as mean ± SEM. Statistical analyses were determined by unpaired two-tailed Student’s *t*-test (**B**-**J**, **L**–**R**).

Browning or whitening is highly correlated with thermogenesis of BAT which is essential for maintaining body temperature and counteracting cold exposure [2]. To investigate the role of SOX4 in thermogenic function, we first analyzed the surface temperature of newborns of *Sox4-MKO* and control littermates on postnatal day 1 (Fig. 2A, B). Infrared imaging showed that the surface temperature in *Sox4-MKO* mice (28.18 ± 0.35 °C) was remarkably lower than that of control littermates (30.15 ± 0.14 °C). Next, 8-week *Sox4*^*f/f*^ and *Sox4-MKO* male mice were exposed to either room temperature (25 °C) or 4 °C for 4 h, and core temperature was measured. As shown in Fig. 2C, both groups exhibited similar core temperatures (38 °C) when maintained at room temperature. However, after 4 h cold exposure at 4 °C, core temperature of *Sox4*^*f/f*^ mice was maintained at about 36.4 °C while that of *Sox4-MKO* mice declined to 34.7 °C. Since BATs primarily utilize glucose and fatty acids from plasma to generate heat in response to acute cold exposure, we measured blood glucose and free fatty acid (FFA) levels. As shown in Fig. 2D and E, *Sox4-MKO* mice exhibited significantly lower blood glucose levels and higher FFA levels at 4 °C, indicating a preference for glucose consumption while exhibiting impaired fatty acid utilization under acute cold exposure. And the triglyceride (TG) levels of BATs in *Sox4-MKO* mice were higher than those in *Sox4*^*f/f*^ mice at both room temperature and 4 °C, with acute cold exposure not exacerbating this difference (Fig. 2F). Additionally, the consumption of O_2_ and heat production of *Sox4-MKO* mice were significantly lower than that of *Sox4*^*f/f*^ mice when mice were subjected into 4 °C (Fig. 2G, H). We then used CL316, 243, a β3-adrenergic receptor agonist, to mimic the effect of cold stimulation. The increase in oxygen consumption and heat production induced by CL316, 243 treatment was significantly attenuated in *Sox4-MKO* mice compared to *Sox4*^*f/f*^ mice (Fig. 2I, J). As skeletal myocytes also arise from *Myf5*^*+*^ progenitor cells and function in shivering thermogenesis under cold stimulation [27], we examined muscle phenotype in *Sox4-MKO* mice but no apparent defects were observed (Fig. S2). Additionally, the role of SOX4 in BAT thermogenesis was further assessed using BAT-specific *Sox4* knockout mice. Similar to *Sox4-MKO* mice, both newborns (Fig. 2K, L) and adults (Fig. 2M–R) of *Sox4-BKO* mice exhibited decreased body temperature and impaired thermogenesis upon acute cold exposure. These results confirmed that SOX4 is required for thermogenic function of BAT.

To further investigate the role of SOX4 in facilitating thermogenesis of BAT, we characterized the phenotype of BAT from preadipocyte-specific *Sox4* transgenic mice (*Pref1-Sox4*). With higher expression of SOX4, BATs from *Pref1-Sox4* mice showed a deeper brown color and smaller fat droplets (Fig. S3A–C). And the surface temperatures of newborn WT and *Pref1-Sox4* pups (P1) were measured using infrared imaging following a 30-minute exposure to room temperature. The surface temperature of *Pref1-Sox4* pups was significantly higher than that of WT pups (Fig. S3D, E). Furthermore, 8-week-old male WT and *Pref1-Sox4* mice were subjected into a cold challenge at 4 °C for 6 hours. The body temperature of *Pref1-Sox4* mice declined slower than that of WT mice (Fig. S3F). After 6 hours of cold exposure, the body temperature reached 35.42 ± 0.34 °C in WT mice while it remained at a higher level of 37.04 ± 0.42 °C in *Pref1-Sox4* mice (Fig. S3F). Additionally, *Pref1-Sox4* mice exhibited increased glucose consumption and triglyceride utilization compared to WT controls (Fig. S3G, H). And metabolic cage analysis revealed a significant increase in O_2_ consumption and heat production in *Pref1-Sox4* mice with acute cold stimuli (Fig. S3I, J). The adeno-associated virus (AAV) system with *Ucp1* mini-promoter and enhancer was used to deliver GFP (*AAV-GFP*) or SOX4 (*AAV-SOX4*) into 6-week-old male mice via tail vein injection (Fig. S3K, L). BAT from *AAV-SOX4* mice had deeper brown color and smaller adipocyte size (Fig. S3M, N), and their body temperatures were significantly higher than control mice by approximately 1 °C after 4 h cold exposure (Fig. S3O). Additionally, *AAV-SOX4* mice showed elevated heat production compared to *AAV-GFP* mice after cold stimulation (Fig. S3P). The data above suggested that overexpression of SOX4 enhances heat production and thermogenesis of BAT.

### Loss of SOX4 in BAT promotes HFD-induced obesity

**Fig. 3:**
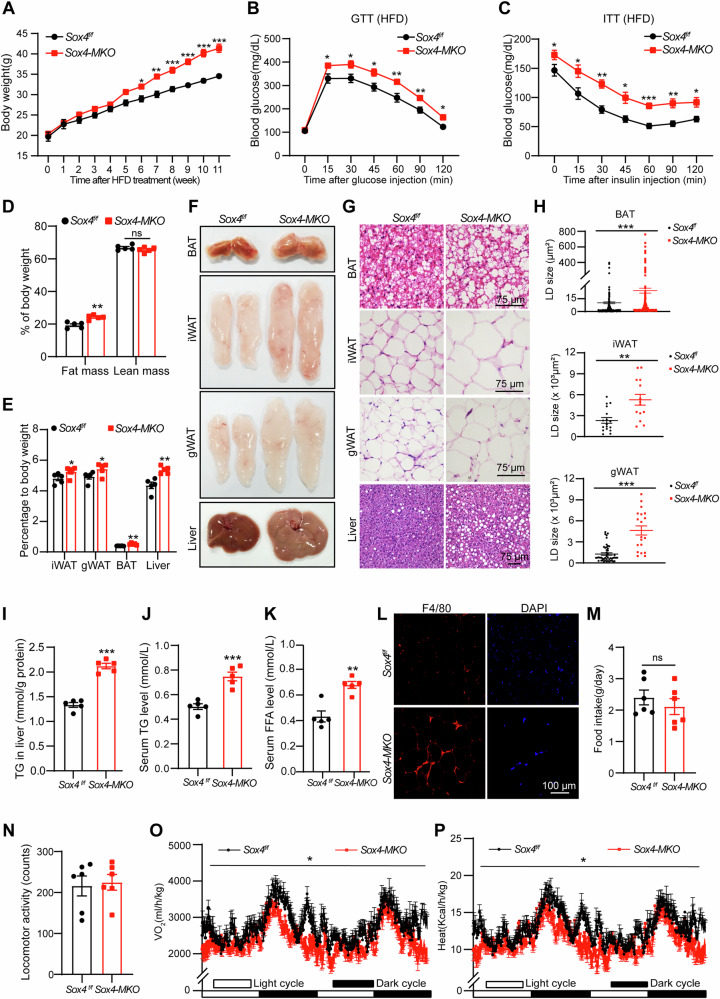
Loss of SOX4 in BAT promotes HFD-induced obesity. **A** The 6-week-old male *Sox4*^*f/f*^ and *Sox4-MKO* mice (*Sox4*^*f/f*^, n = 5; *Sox4-MKO*, n = 6) were subjected to HFD feeding. Body weight was monitored every week. **B** On week 9, mice were fasted for 16 h and then received an intraperitoneal injection of D-glucose (1.5 mg/kg) for glucose tolerance test. Blood glucose levels were measured from tail vein at indicated time (n = 5). **C** On week 11, mice were fasted for 6 h and then received an intraperitoneal injection of insulin at a dose of 1 U/kg for an insulin tolerance test. Blood glucose levels were measured from tail vein at indicated time (n = 5). **D** On week 9, the average fat and lean mass of *Sox4*^*f/f*^ *and Sox4-MKO* male mice were measured using Echo MRI composition analyzer (n = 5). **E**–**H** At the end of HFD feeding period, mice were sacrificed. The BAT, iWAT, gWAT, liver and blood were collected. The ratios of tissue weight/body weight were plotted in (**E**) (n = 5). Representative appearance (**F**) and H&E staining (**G**) were shown. Scale bar, 75 μm.The sizes of lipid droplets from the H&E staining images (**G**) were quantified using ImageJ (**H**). The TG content of liver was measured (**I**) (n = 5). **J**, **K** Serum levels of TG (**J**) and FFA (**K**) were measured (n = 5). **L** Immunofluorescence (IF) analysis for the F4/80 expression in gWAT from HFD-fed mice, Scale bar, 100 μm. **M**–**P** On week 11, the HFD-fed mice were subjected to metabolic cage analysis. Food intake (**M**), locomotor activity (**N**), oxygen consumption (**O**) and heat production (**P**) of mice were measured in 2 consecutive days (n = 6). Asterisks (*) denote the level of statistical significance. ns, no significance, *p < 0.05; **p < 0.01; ***p < 0.001. Data are presented as mean ± SEM. Statistical analyses were determined by unpaired two-tailed Student’s *t*-test (**A**–**E**, **H**, middle panel; **I**–**K**, **M**–**P**) and unpaired two-tailed Mann-Whitney test (**H**, top panel and bottom panel).

Non-shivering thermogenesis of BAT expends energy, contributing to the maintenance of whole-body metabolic homeostasis. We investigated whether the absence of SOX4 in BAT contributes to obesity development. 6-week-old male mice with *Sox4*^*f/f*^ and *Sox4-MKO* genotypes were fed either a chow diet (Fig. S4) or a high-fat diet (HFD) (Fig. 3). After 6 weeks of HFD feeding, the body weight of *Sox4-MKO* mice significantly exceeded that of *Sox4*^*f/f*^ mice (Fig. 3A), while on a normal chow diet, both groups exhibited similar weight gain patterns (Fig. S4A). Furthermore, HFD-fed *Sox4-MKO* mice displayed more severe glucose intolerance and insulin resistance than their control counterparts (Fig. 3B, C). Body composition analysis revealed an increase in body fat, while the lean composition remained unchanged in HFD-fed *Sox4-MKO* mice compared to the controls (Fig. 3D). Consistently, the percentages of BAT, iWAT, gWAT, and liver were markedly higher in HFD-fed *Sox4-MKO* mice compared to those in controls (Fig. 3E), with larger individual fat pads and livers observed as well (Fig. 3F). Histological analysis showed significantly larger lipid droplets in BAT, iWAT, and gWAT of HFD-fed *Sox4-MKO* mice, along with increased lipid accumulation in liver sections (Fig. 3G, H). Moreover, the liver TG content, serum TG levels, and FFA levels were significantly elevated in *Sox4-MKO* mice fed a HFD (Fig. 3I–K). Consistent with an increased susceptibility to obesity, F4/80, a macrophage marker, was found to be significantly upregulated in the gWAT of HFD-fed *Sox4-MKO* mice compared to their *Sox4*^*f/f*^ counterparts (Fig. 3L). Metabolic cage analysis further confirmed that HFD-fed *Sox4-MKO* mice displayed significantly reduced oxygen consumption and heat production, while food intake and locomotor activity remained similar to those of *Sox4*^*f/f*^ mice (Fig. 3M–P). On normal chow diet (NCD), the mass and the morphology of BAT, iWAT, gWAT and liver, as well as O_2_ consumption and heat production, were similar between *Sox4-MKO* and *Sox4*^*f/f*^ mice (Fig. S4B–H). These data suggest that loss of SOX4 in BAT impairs thermogenesis and reduces energy consumption, thereby increasing susceptibility to obesity when exposed to HFD.

We further investigated whether overexpression of SOX4 in BAT could counteract HFD-induced obesity. Mice injected with *AAV-GFP* or *AAV-SOX4* were fed with NCD or HFD (Fig. S5A). On NCD, both groups exhibited similar weight gain (Fig. S5B). After 5 weeks on HFD, the body weight of *AAV-SOX4* mice was significantly lower than that of *AAV-GFP* mice (Fig. S5B). After 10 weeks of HFD treatment, *AAV-SOX4* mice displayed improved blood glucose tolerance and insulin sensitivity, and reduced body fat composition (Fig. S5C–E). The percentages of BAT, iWAT, gWAT and liver in *AAV-SOX4* mice were significantly lower than those in *AAV-GFP* mice (Fig. S5F). Additionally, *AAV-SOX4* mice had decreased serum TG and FFA levels (Fig. S5G, H). Consistently, compared to the control mice, *AAV-SOX4* mice had smaller individual fat pads and liver (Fig. S5I). H&E staining showed that *AAV-SOX4* mice had markedly smaller lipid droplets in BAT, iWAT, gWAT and liver (Fig. S5J, K). Metabolic cage analysis revealed increased oxygen consumption and heat production in *AAV-SOX4* mice while food intake and locomotor activity remained similar to those of *AAV-GFP* mice (Fig. S5L–O). Overall, these results demonstrate that overexpressing SOX4 in BAT increases energy expenditure and attenuates HFD-induced obesity.

### SOX4 promotes BAT-selective genes expression in vivo

**Fig. 4:**
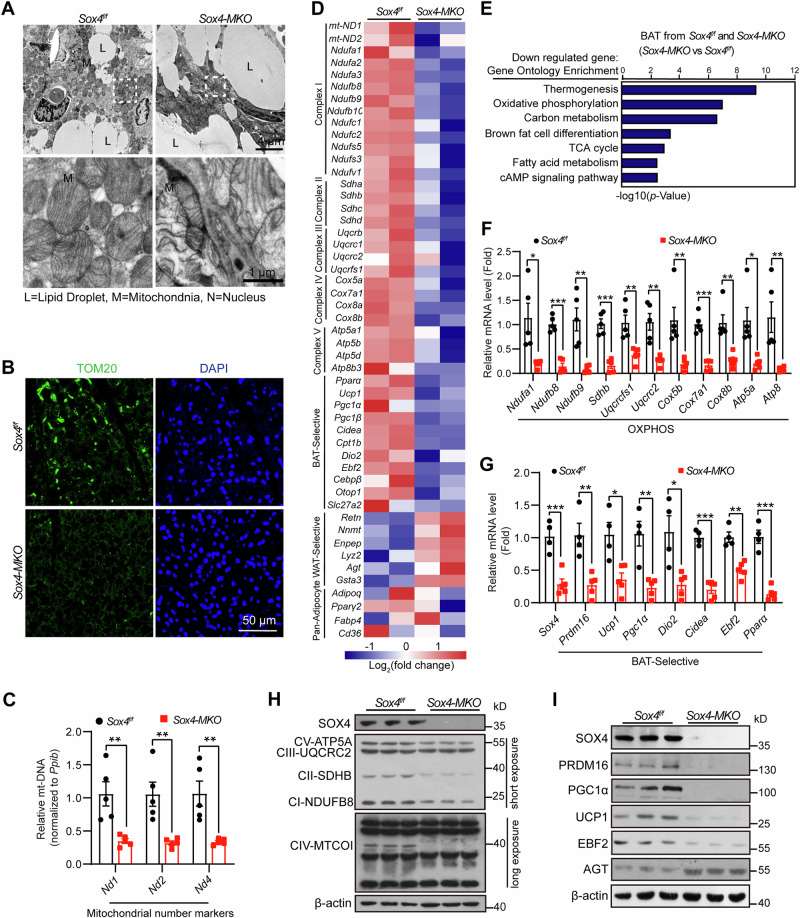
Deletion of SOX4 impairs expression of BAT-selective genes in vivo. **A** Transmission electron micrograph of BAT from 4-week-old male *Sox4-MKO* and control littermates (LD, lipid droplet; M, mitochondria; N, nucleus). Scale bars are as indicated. **B** Immunofluorescence (IF) analysis for the TOM20 expression in BAT of 10-week-old male mice. Scale bar, 50 μm. **C** Mitochondrial-specific transcripts (*Nd1*, *2* and *4*) were measured by qPCR in BAT mitochondrial DNA (*mt-DNA*) of 9-week-old *Sox4-MKO* and control male mice (n = 5). *Ppib* was used as an invariant control. The mRNA levels in control mice were normalized to 1.0. **D**, **E** *Sox4*^*f/f*^ *and Sox4-MKO* male mice (15-week-old) were sacrificed and BAT tissue were collected for RNA-seq analysis. Heat map analysis of the representative genes (**D**). The down-regulated genes were used for clustering analysis and were plotted in (**E**). **F** RT-PCR analysis of mitochondrial complex genes in BAT of 9-week-old male *Sox4-MKO* and control littermates (n = 5). *18S* was used as an invariant control. The mRNA levels in control mice were normalized to 1.0. **G** RT-PCR analysis of the representative BAT-selective gene in BAT of 9-week-old male *Sox4-MKO* and their respective control mice (*Sox4*^*f/f*^, n = 4; *Sox4-MKO*, n = 5). *18S* was used as an invariant control. The mRNA levels in control mice were normalized to 1.0. **H** Western blot analysis of SOX4 and mitochondrial respiratory chain components in BAT from 9-week-old male *Sox4-MKO* and control mice (n = 3). **I** Western blot analysis of BAT-selective proteins and AGT protein in BAT from 9-week-old male *Sox4-MKO* and control mice (n = 3). Asterisks (*) denote the level of statistical significance. *p < 0.05, **p < 0.01, ***p < 0.001. Data are presented as mean ± SEM. Statistical analyses were determined by unpaired two-tailed Student’s *t*-test (**C,** **F,** **G**).

We then investigated how SOX4 regulates BAT development and thermogenesis. The brown coloration of BAT is attributed to its abundant presence of mitochondria and cytochrome, which confers a rich metabolic capacity [28]. In S*ox4-MKO* mice, we observed a whitening phenomenon in BAT, suggesting potential alterations in mitochondrial structure and function. Transmission electron microscopy analysis showed that brown adipocytes from *Sox4-MKO* mice exhibited larger lipid droplets and disorganized cristae within their mitochondria compared to control mice (Fig. 4A). Furthermore, quantitative analysis revealed a decrease in both the number of mitochondria and mitochondrial DNA content in BAT from *Sox4-MKO* mice (Fig. 4B, C).

To further elucidate the impact of SOX4 on BAT development, we performed RNA sequencing of BAT tissues from *Sox4*^*f/f*^ and *Sox4-MKO* mice. Heatmaps revealed downregulation of numerous genes involved in mitochondria complex I-V, as well as key BAT-selective genes including *Pparα*, *Ucp1, PGC1α*, *Cidea, Cpt1b, Dio2, Ebf2* in the BAT of *Sox4-MKO* mice (Fig. 4D). Gene ontology (GO) analysis revealed the downregulated genes in the *Sox4-MKO* mice were associated with thermogenesis, mitochondrial oxidative phosphorylation, brown fat cell differentiation, TCA cycle and fatty acid metabolism (Fig. 4E). These findings were further validated through qPCR analysis (Fig. 4F, G). PGC1α, a crucial regulator of mitochondrial biogenesis, is essential for maintaining BAT thermogenesis [29]. The low expression of PGC1α in BAT of *Sox4-MKO* mice suggested a potential link between SOX4 deficiency, impaired mitochondria, and compromised thermogenesis. Moreover, levels of mitochondrial complex proteins and BAT-selective proteins were reduced in BAT from *Sox4-MKO* mice (Fig. 4H, I). Notably, we observed a significant upregulation of WAT-selective genes in BAT of *Sox4-MKO* mice (Fig. 4D, and Fig. S6A), while the expression levels of common pan-adipocyte markers remained comparable to those in *Sox4*^*f/f*^ mice (Fig. S6B). Consistent with the whitening of BAT, the mRNA levels of lipolysis-related genes *Hsl*, *Atgl*, and *Mgll* were significantly downregulated in *Sox4-MKO* mice (Fig. S6C). Additionally, reduced levels of p-HSL/HSL and phospho-PKA substrates, along with elevated PLIN1 levels in the BAT further indicated suppressed lipolysis in *Sox4-MKO* mice (Fig. S6D, E). Moreover, the whitening of BAT indicated increased inflammation and lipogenesis [30, 31]. Consequently, we reanalyzed the RNA-seq data from BATs of *SOX4*^*f/f*^ and *Sox4-MKO* mice with GO analysis, which revealed a significant upregulation of genes associated with inflammation and lipogenesis (Fig. S6F, G). These findings were further confirmed by qPCR (Fig. S6H, I).

We also performed a rescue experiment by reintroducing SOX4 into *Sox4-MKO* mice via adenovirus injection (Fig. S7A). 7 days after the first injection, BAT tissues were collected for analysis. qPCR analysis revealed that the expressions of BAT-selective genes, including *Ucp1*, *Prdm16*, *Pgc1α*, and *Dio2*, were fully restored in BAT expressing SOX4 (Fig. S7B). Immunofluorescence staining further confirmed protein levels of UCP1 and PRDM16 were restored with SOX4 expression (Fig. S7C). Additionally, histological analysis showed that SOX4 overexpression rescued the enlarged lipid droplets observed in BAT of *Sox4-MKO* mice (Fig. S7D, E). We also explored whether overexpression of SOX4 could promote the expression of BAT-selective genes. We observed increased expression of BAT-specific genes and decreased expression of white-specific genes in *AAV-SOX4* mice (Fig. S7F, G). Immunohistochemical experiments further confirmed that *AAV-SOX4* mice exhibited enhanced UCP1 expression compared to *AAV-GFP* mice (Fig. S7H). Collectively, these findings demonstrate that SOX4 is essential for the expression of BAT-selective genes which are crucial for maintain BAT characteristics.

### SOX4 is required for brown adipocyte differentiation in vitro

**Fig. 5:**
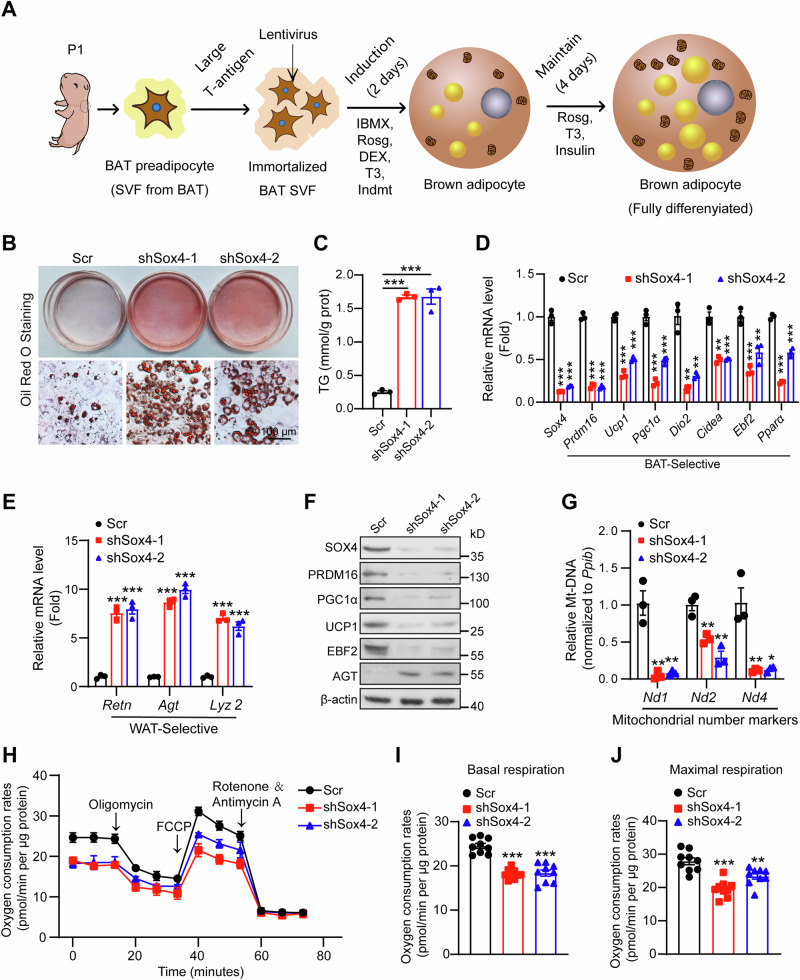
SOX4 is required for brown adipocyte differentiation in vitro. **A** Schematic drawing showed that the immortalized BAT SVF cells were established and differentiated into mature adipocytes. **B,** **C** The immortalized BAT SVF cells infected with scrambled or shSox4 lentivirus were differentiated into mature adipocytes as indicated in Fig. 5A. On day 6 of differentiation, mature brown adipocytes were subjected to Oil Red O staining (**B**) and triglyceride measurement (**C**) (n = 3). Scale bar, 100 μm. **D**, **E** The mRNA levels of BAT-selective genes (**D**) and WAT-selective genes (**E**) in differentiated BAT adipocytes generated as in (**A**) (n = 3). *18S* was used as an invariant control. The mRNA levels in control cells were normalized to 1.0. **F** The protein levels of BAT enriched proteins and AGT in mature brown adipocytes. **G** RT-PCR analysis of mitochondrial number markers in mature brown adipocytes (n = 3). *Ppib* was used as an invariant control. The mRNA levels in control cells were normalized to 1.0. **H**–**J** BAT SVF cells were infected with scrambled or shSox4 lentivirus and analyzed for Oxygen consumption rate (OCR) at day 6 of differentiation. Oligomycin, FCCP, and Rotenone / Antimycin were added at the time points indicated by the arrows and OCR was showed in (**H**). The averaged basal and maximal respiration rates were shown in (**I**) and (**J**), respectively (n = 3). Asterisks (*) denote the level of statistical significance. *p < 0.05, **p < 0.01, ***p < 0.001. Data are presented as mean ± SEM. Statistical analyses were determined by unpaired two-tailed Student’s *t*-test (**C**–**E**, **G**–**J**).

The differentiation of brown adipocytes and the development of BAT critically rely on genes selectively expressed in BAT [32, 33]. To investigate the essential role of SOX4 in brown adipocyte differentiation in vitro, we immortalized BAT SVF cells isolated from newborn male WT mice for subsequent experiments. The immortalized BAT SVF cells were infected with lentivirus expressing scramble or shSox4 and then induced to differentiate into mature brown adipocytes in vitro (Fig. 5A). Oil Red O staining revealed that the loss of SOX4 promoted lipid storage in mature brown adipocytes, resulting in significantly elevated triglyceride levels (Fig. 5B, C). The expression of BAT-selective genes was significantly downregulated, while WAT-selective genes were upregulated (Fig. 5D, E). Western blot analysis showed that the BAT-selective protein levels in SOX4 knockdown adipocytes were noticeably decreased, whereas the protein levels of AGT were significantly increased (Fig. 5F). Additionally, mitochondria-containing DNA was largely depleted in SOX4 knockdown cells (Fig. 5G). Consistently, SOX4 knockdown cells displayed reduced levels of basal mitochondrial respiration and maximal mitochondrial respiratory capacity (Fig. 5H–J). Similar results were observed in brown adipocytes differentiated from primary BAT SVFs isolated from *Sox4-MKO* mice (Fig. S8A–J). These results demonstrate that SOX4 is essential for brown adipocytes differentiation in vitro.

### SOX4 activates transcription of EBF2 facilitating BAT development

**Fig. 6:**
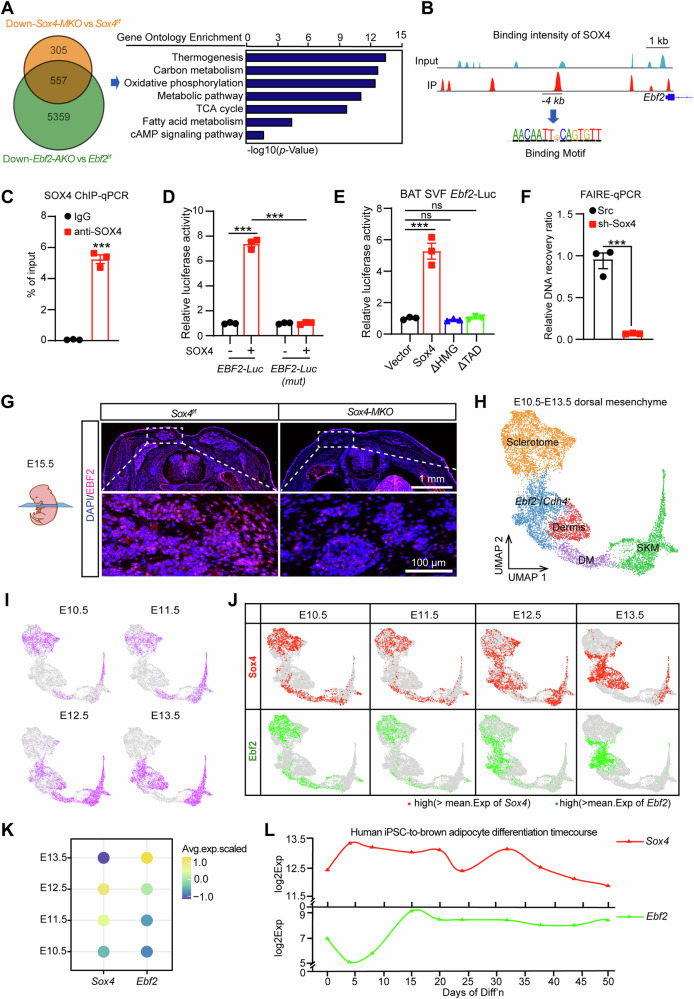
SOX4 activates transcription of EBF2 facilitating BAT development. **A** RNA-seq data from *Sox4-MKO* BAT with that from *Ebf2-AKO* BAT (GSE144188). The overlapping down-regulated genes were subjected to gene ontology analysis (right). **B** ChIP-seq revealed a significantly enriched binding of SOX4 at -4k upstream of the Ebf2 promoter. **C** ChIP analysis showing SOX4 protein occupancy at -4k upstream of the Ebf2 promoter in BAT SVF (n = 3). **D** Luciferase activity of wild-type or SOX4-binding site mutant pGL4.26-EBF2 vectors in NIH3T3 cells transfected with or without SOX4 (n = 3). **E** Relative transcriptional activity of the *Ebf2* promoter in NIH3T3 cells with expression of vector, SOX4, ΔHMG and ΔTAD (n = 3). **F** BAT SVF cells were infected with scrambled or shSOX4 lentiviruses for 2 days and then subjected to FAIRE assay. The enriched DNAs were examined by qPCR for the *Ebf2* promoter (n = 3). **G** Immunofluorescence (IF) analysis for the EBF2 expression in E15.5 *Sox4*^*f/f*^ and *Sox4-MKO* embryos. Scale bars are as indicated. **H** UMAP of 10,163 mesenchymal and skeletal muscle cells from E10.5 to E13.5 mouse embryos, indicating clusters expected to be found in the dorsal region of the mouse embryo, where BAT develops. **I** Cells in (**H**) highlighted by developmental stage of origin. **J** *Sox4* and *Ebf2* expressions in different cell clusters from E10.5 to E13.5. **K** Dot plot shows the average expression level of *Ebf2* and *Sox4* from E10.5 to E13.5 mouse embryos. **L** The mRNA expression levels of *Sox4* and *Ebf2* during the differentiation of human iPSCs into brown adipocytes. Asterisks (*) denote the level of statistical significance. ns, no significance, ***p < 0.001. Data are presented as mean ± SEM. Statistical analyses were determined by unpaired two-tailed Student’s *t*-test (**C,** **F**), one-way ANOVA followed by Tukey’s test (**E**) and two-way ANOVA followed by Tukey’s test (**D**).

To elucidate the mechanism by which SOX4 regulates brown adipocyte differentiation, we examined the expression of adipogenic regulators at different time points. Notably, expression of *Ebf2* was remarkably decreased in Sox4-knockdown cells at the early stage of differentiation (Fig. S9A, B). Consistently, overexpression of SOX4 promoted the expression of EBF2 (Fig. S9C). We further compared RNA-seq data from *Sox4-MKO* BAT with that from *Ebf2-AKO* BAT (GSE144188) (Fig. 6A), and we found that 862 genes were down-regulated in *Sox4 MKO* mice (KO: WT < 0.05). Among these genes, 557 overlapped with the down-regulated genes identified in *Ebf2 AKO* mice. GO analysis revealed that the overlapping downregulated genes were associated with thermogenesis, oxidative phosphorylation, brown fat cell differentiation, TCA cycle and fatty acid metabolism (Fig. 6A). These indicates that SOX4 may regulate BAT development and thermogenesis via EBF2. To further confirm this hypothesis, we isolated primary BAT SVFs from *Sox4*^*f/f*^ and *Sox4-MKO* mice and performed a rescue experiment in SOX4-knockout BAT SVFs. As depicted in Fig. S9D, E, overexpression of EBF2 significantly reduced TG content and restored the expression of BAT-selective genes in SOX4-knockout cells. Furthermore, knockdown of EBF2 largely blocked enhancement of BAT-selective genes expression mediated by overexpression of SOX4 (Fig. S9F). These results suggest that SOX4 promotes the expression of BAT-selective genes by enhancing the transcription of EBF2.

To address the direct regulation of EBF2 transcription by SOX4, we performed ChIP-seq analysis. As shown in Fig. 6B, ~4 kb region of *Ebf2* was bound by SOX4, and ChIP-qPCR assays confirmed specific binding of SOX4 to this region (Fig. 6C). Next, the SOX4 binding region in *Ebf2* was cloned into a pGL4.26 vector for luciferase reporter assay. SOX4 significantly increased the luciferase activity, and this activation could be blocked by mutation of SOX4 binding site (AACAAGT mutant to GCTCGGT) (Fig. 6D). To elucidate whether the transcriptional activation of Ebf2 relies on the HMG-box domain (HMG-BOX DNA-binding domain) or the TAD domain (C-terminal transactivation domain) of SOX4 protein, we generated SOX4 mutants lacking either the HMG-box domain (ΔHMG) or the TAD domain (ΔTAD) (Fig. S9G). Luciferase assay results revealed that when either the HMG-box domain or TAD domain was absent, SOX4 failed to activate Ebf2-luciferase activity (Fig. 6E). Further, FAIRE-qPCR assay showed knockdown of SOX4 markedly reduced chromatin accessibility at *Ebf2* enhancer (Fig. 6F). These results indicate that SOX4 directly activates the transcription of EBF2.

EBF2 determines brown adipocyte identity by recruiting PPARγ to its brown-selective binding sites [15]. We found that EBF2 protein levels in BAT were remarkably decreased in both embryonic and adult *Sox4 MKO* mice (Figs. 4I and 6G), while PPARγ protein levels were not affected by loss of SOX4 (Fig. S9H). Therefore, we investigated whether the loss of SOX4 reduced PPARγ binding to BAT-selective genes. BATs from *Sox4*^*f/f*^ and *Sox4-MKO* mice were subjected into ChIP-qPCR using anti-PPARγ antibody. The loss of SOX4 reduced PPARγ binding at promoter regions of *Ucp1* and *Prdm16*, and increased PPARγ binding at promoter regions of *Agt* (Fig. S9I). Taken together, these results suggested that SOX4 activated EBF2 expression, promoting PPARγ binding to promoters of BAT-selective genes, which facilitates BAT development and thermogenic function.

To assess the role of SOX4 at the early stages of BAT development, we examined control and mutant embryos at E15.5. Immunostaining analysis revealed a significant decrease in EBF2 protein levels in *SOX4-MKO* mice (Fig. 6G). We analyzed expression profiles of SOX4 and EBF2 using scRNA-seq data from the dorsal-anterior region of mouse embryos at E10.5–E13.5 (GSE233955) (Fig. 6H) [34]. The evaluation of the cell profiles at different developmental time points revealed a prominent presence of sclerotome at E10.5-E11.5 and the existence of *Cdh4*^*+*^/ *Ebf2*^*+*^ cell cluster was observed at E12.5 and E13.5 (Fig. 6I). The expression of SOX4 and EBF2 exhibited high degree of spatial coincidence in sclerotome cells and *Ebf2*^*+*^*/Cdh4*^*+*^ BAT preadipocytes at E10.5-E13.5 (Fig. 6J). And the peak expression of SOX4 was observed at E12.5 which is earlier than the peak expression time of EBF2 (Fig. 6K). Furthermore, we analyzed the expression patterns of SOX4 and EBF2 using RNA-Seq data from human induced pluripotent stem cells (iPSCs) that were differentiated to brown adipocytes (GSE131169). In this process, highest expression levels of SOX4 were observed during the early stage, followed by an increase in EBF2 expression (Fig. 6L). Collectively, these results suggest SOX4 plays a regulatory role at early-stage of BAT development.

### Phosphorylation of SOX4 by PKA facilitates its nuclear translocation and enhances the transcription of *Ebf2*

**Fig. 7:**
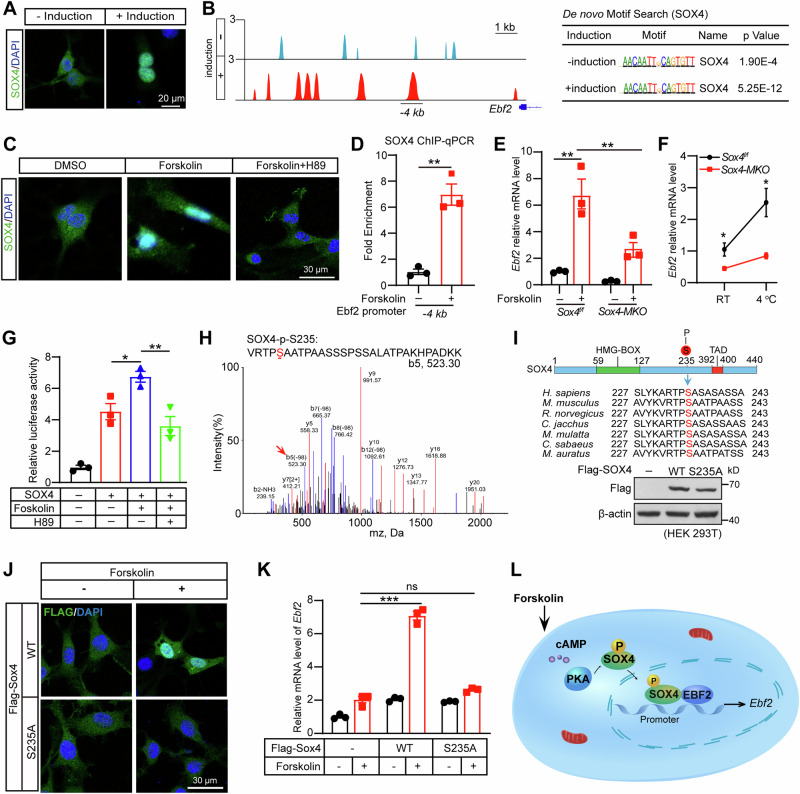
Phosphorylation of SOX4 by PKA facilitates its nuclear translocation and enhances the transcription of EBF2. **A** BAT SVF cells were treated with or without induction medium for 1 h. Then cells were fixed and subjected into immunostaining by using anti-SOX4 antibody. Scale bar, 20 μm. **B** Control and 3X HA-SOX4 expressing BAT SVF cells were treated with or without induction medium for 1 h. Cells were harvested and subjected into chromatin immunoprecipitation by using anti-HA antibody. ChIP-Seq analysis showed binding profiles of SOX4 on the promoter of *Ebf2* and de novo motif analysis of SOX4 binding sites. **C** BAT SVF cells were treated with DMSO, forskolin (20 μM) or H89 (30 μM) prior to stimulation with forskolin (20 μM) for 60 min. After 1 hour of stimulation, we performed immunofluorescence assay to detect the cytoplasmic-nuclear distribution of SOX4 protein. Scale bar, 30 μm. **D** ChIP-qPCR showing *Sox4* occupancy at the promoter of *Ebf2* in BAT SVF treated without or with forskolin (20 μM) for 1 h (n = 3). **E** BAT SVF cells isolated from 3-week-old *Sox4-MKO* and control littermates were cultured to confluence. Cells were treated with or without forskolin (20 μM) for 1 h and then harvested for RT-qPCR analysis (n = 3). *18S* was used as an invariant control. The mRNA levels in control cells were normalized to 1.0. **F** *Sox4*^*f/f*^ and *Sox4-MKO* mice (8-week-old male, n = 4) were housed at RT or 4 °C for 4 h. Subsequently, mice were dissected, and BAT was collected. BAT SVFs were isolated from BAT for qPCR analysis of *Ebf2*. **G** BAT SVF reaching indicated confluence were subjected to different supplement (PBS, forskolin or H89) as described in (**C**). Transcriptional activity of the Ebf2 enhancer in NIH3T3 were analyzed by luciferase reporter assay (n = 3). **H** Mass spectrometry analysis of phosphorylation sites on SOX4. HEK293T cells were transfected with WT-PRKACA (catalytic subunit of PKA) or a kinase-dead mutant of PKA (KD-PRKACA) together with HA-SOX4 for 48 h. The cells were lysed and subjected to immunoprecipitation (IP) against HA, then the pellet was separated on SDS-PAGE and subjected to mass spectrometry analysis. **I** Alignment of SOX4 residues S325, along with the flanking amino acid residues from different species. HEK293T cells were transfected with WT or S235A mutant of SOX4. After 48 hours, cells were harvested and analyzed by western blot (n = 3). **J**, **K** NIH3T3 were transfected with Flag-SOX4 (WT) or S235A mutant. 36 h later, cells were treated with DMSO or forskolin (20 μM) for 1 h. And then cells were fixed for immunofluorescence assay (**J**) (Scale bar, 30 μm) or collected for qPCR analysis (**K**) (n = 3). **L** The illustration showed that forskolin induces PKA activation, leading to the phosphorylation of SOX4 at S235 and its subsequent translocation into the nucleus. Once in the nucleus, SOX4 cooperates with EBF2 to activate transcription of EBF2. Asterisks (*) denote the level of statistical significance. *p < 0.05, **p < 0.01, ***p < 0.001. Data are presented as mean ± SEM. Statistical analyses were determined by unpaired two-tailed Student’s *t*-test (**D**, **F**), one-way ANOVA followed by Tukey’s test (**G**) and two-way ANOVA followed by Tukey’s test (**E,** **K**).

Notably, induction medium could promote nuclear localization of SOX4 in brown adipocytes differentiation (Fig. 7A). We also performed ChIP-Seq to visualize the binding of SOX4 to *Ebf2* enhancer in the presence or absence of induction medium. As shown in Fig. 7B, SOX4 directly bound to -4 kb region of *Ebf2*, and its binding intensity was dramatically increased by treatment of induction medium. The administration of forskolin could mimic the effect of induction medium on SOX4 nuclear translocation and its binding to *Ebf2* enhancer, thereby increased the mRNA levels of *Ebf2* (Fig. 7C–E). Notably, the loss of SOX4 significantly blunted the increase in *Ebf2* mRNA levels by forskolin treatment (Fig. 7E). Cold stimulation can increase the population of brown precursor fat cells [35]. EBF2 specifically marks and regulates the molecular profile of brown preadipocytes [14]. Therefore, *Sox4*^*f/f*^ and *Sox4-MKO* mice were subjected into RT or 4 °C for 4 h, and primary BAT SVFs were isolated for qPCR analysis. As shown in Fig. 7F, the increased mRNA levels of *Ebf2* induced by cold stimuli were largely blunted in the BAT of *Sox4-MKO* mice. The PKA inhibitor H89 effectively blocked the nuclear translocation of SOX4 and SOX4-mediated transcriptional activation of EBF2 induced by forskolin (Fig. 7C, G). Subsequently, HA-SOX4 was purified from cells overexpressing either PKACA or a dominant negative variant of PKACA, followed by comprehensive mass spectrometry analysis. The phosphorylated S235-containing peptide of SOX4 was specifically detected in PKACA expressing cells, as illustrated in Fig. 7H. Additionally, sequence alignment analysis revealed the conservation of residue S235 in SOX4 across diverse species (Fig. 7I). To investigate the impact of S235 phosphorylation, we generated an S235A mutant to mimic unphosphorylated SOX4 (Fig. 7I). This mutant was unable to enter the nucleus and failed to activate *Ebf2* transcription upon forskolin stimulation (Fig. 7J, K). Taken together, these findings suggest that forskolin induces PKA activation, leading to the phosphorylation of SOX4 at S235 and its subsequent translocation into the nucleus (Fig. 7L).

### SOX4 cooperates with EBF2 to activate the transcription of *Ebf2*

Considering the adjacent binding sites of SOX4 and EBF2 at the region of *Ebf2* (Fig. S10A), we sought to investigate whether these two factors could collaborate in activating EBF2 transcription. First, SOX4 and EBF2 could be coprecipitated and exhibited nuclear colocalization in BAT SVF cells (Fig. S10B–D). Notably, luciferase experiments showed that individual expression of SOX4 or EBF2 led to about a 5-fold activation, whereas their combined effect synergistically enhanced EBF2 transcription approximately 33-fold (Fig. S10E). We further explored the SOX4-EBF2 interaction interfaces, revealing that the TAD domain of SOX4 is required for its interaction with EBF2 (Fig. S10F, G). Moreover, the truncation lacking either the HMG or TAD domain did not exhibit a synergistic effect with EBF2 in enhancing EBF2 transcription (Fig. S10H), thereby suggesting that the synergistic effect between SOX4 and EBF2 relies on both the binding of the HMG domain to the promoter region and the interaction of TAD domain with EBF2. These results reveal that SOX4 could cooperate with EBF2 to activate transcription of EBF2 (Fig. 7L).

### SOX4 cooperates with EBF2 to enhance expression of thermogenic genes

**Fig. 8:**
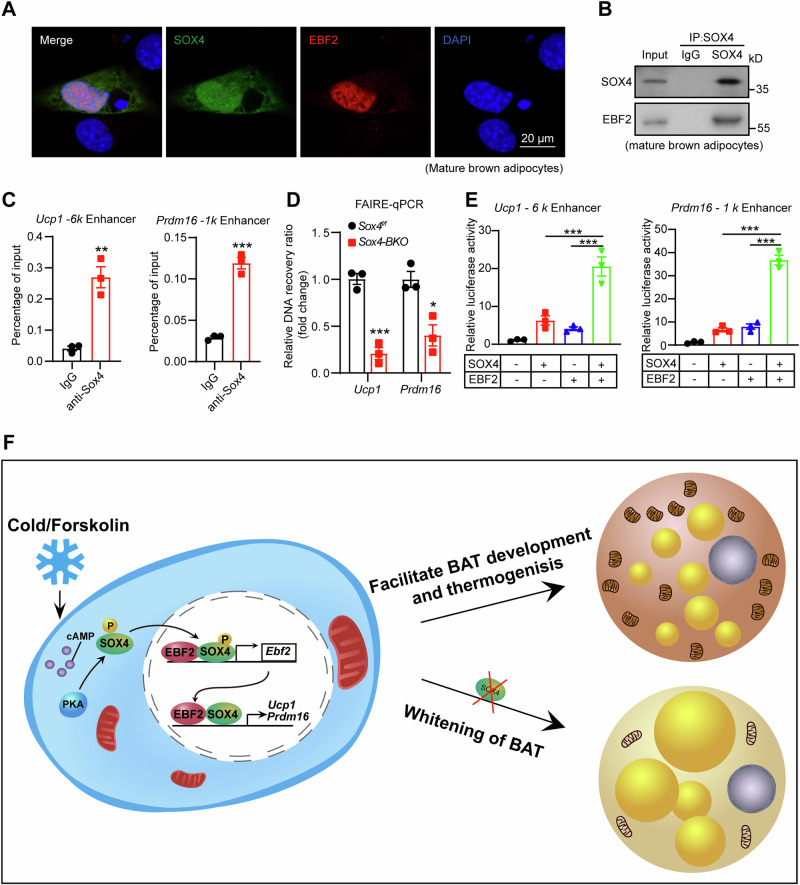
SOX4 cooperates with EBF2 to enhance expression of thermogenic genes. **A** Primary BAT SVFs were isolated from BAT of 8-week-old male WT mouse and then subjected to differentiation. On day 6 of differentiation, cells were harvested and subjected to immunofluorescence analysis. Immunofluorescence analysis showed the co-localization of SOX4 and EBF2 in the nucleus of mature BAT adipocyte. Scale bar, 20 μm. **B** BAT SVF cells were differentiated for 6 days, followed by lysis and immunoprecipitation using anti-SOX4 antibody or IgG. Input and pellet fractions were subsequently analyzed by western blot using the indicated antibodies. **C** BAT SVF cells expressing Flag-SOX4 were subjected to differentiation for 6 days. Following this, cells were harvested, and ChIP assay was performed with the FLAG antibody. The occupancy of SOX4 at -6k region of *Ucp1* reporter and-1k region of *Prdm16* reporter were analyzed by qPCR (n = 3). **D** The BAT SVFs isolated from 3-week-old *Sox4-BKO* and control mice were differentiated into mature adipocytes. On day 6 of differentiation, cells were subjected to the FAIRE assay, and the enriched DNAs were examined by qPCR for the site -5851 upstream of the *Ucp1* promoter or the site -885 upstream of the *Prdm16* promoter (n = 3). **E** Transcriptional activity of the - 6 kb upstream of *Ucp1* promoter and - 1 kb upstream of *Prdm16* promoter was assessed in NIH3T3 cells upon overexpression of SOX4, EBF2, or both (n = 3). **F** A schematic model showing that cold stimuli or forskolin treatment induces the activation of PKA, which subsequently phosphorylates SOX4 to facilitate its translocation into the nucleus. Within the nucleus, SOX4 collaborates with EBF2 to enhance transcription of both EBF2 and downstream thermogenic genes. Asterisks (*) denote the level of statistical significance. *p < 0.05, **p < 0.01, ***p < 0.001. Data are presented as mean ± SEM. Statistical analyses were determined by unpaired two-tailed Student’s *t*-test (**C**, **D**), and one-way ANOVA followed by Tukey’s test (**E**).

Given our clear elucidation of the interaction between SOX4 and EBF2 in promoting EBF2 transcription, we aimed to investigate their potential cooperative role in enhancing the transcription of thermogenic genes targeted by EBF2. SOX4 exhibited colocalization with EBF2 and could precipitate with EBF2 in mature brown adipocytes (Fig. 8A, B). Next, we reanalyzed the ChIP-seq profiles of EBF2 (GSE97114) at the *Ucp1* and *Prdm16* enhancers, which revealed binding peaks of EBF2 in the -6k region of *Ucp1* and -1k region of *Prdm16* [36], which coincided with the conserved binding site for SOX4 (AACAAAG) in these regions (Fig. S11A). Furthermore, ChIP-qPCR experiments confirmed the specific binding of SOX4 to these regions (Fig. 8C). FAIRE-qPCR experiments indicated that loss of SOX4 significantly reduced chromatin accessibility at both *Ucp1* and *Prdm16* (Fig. 8D). Additionally, the luciferase assays demonstrated a significant augmentation in the transcription of *Ucp1* and *Prdm16* when SOX4 and EBF2 were co-overexpressed (Fig. 8E). The SOX4 truncation lacking either the HMG or TAD domain failed to cooperate with EBF2 in activating the transcription of *Ucp1* and *Prdm16* (Fig. S11B, C). These findings collectively suggest that the cooperation between SOX4 and EBF2 drives the transcription of thermogenic genes in brown adipocytes (Fig. 8F).

## Discussion

BAT is essential for non-shivering thermogenesis. It develops at the embryonic stage and is quite important for maintaining body temperature especially for newborns to counteract cold [37]. Promoting BAT development and thermogenic function to elevate energy consumption is a potential strategy to counteract obesity and obesity-related diseases [4]. EBF2 and PRDM16 are identified as key transcription factors for activation of thermogenic gene program to determine brown adipocyte cell fate [6, 15]. In the process of brown adipocyte differentiation, EBF2 expression was stimulated at early stage while PRDM16 expressed at late stage [34, 38]. EBF2, which is highly expressed in BAT, could recruit PPARγ binding to BAT-specific genes and activate BAT-selective genes expression [15]. This is fundamental for brown adipocytes development. However, the upstream regulator of EBF2 remains unknown. Here, we found that deletion of SOX4 impaired EBF2 expression in BAT, thereby suppressing expression of BAT-selective genes and thermogenic genes such as UCP1 and PRDM16. In vitro, the brown adipocytes differentiated from SOX4 knockdown BAT SVF cells exhibited reduced expression of BAT thermogenic genes, leading to the accumulation of large lipid droplets. In contrast, SOX4 overexpression enhanced BAT thermogenic genes expression and reduced the size of lipid droplets. In vivo, similar to EBF2-promoter mice [15, 36], *Sox4 MKO* and *Sox4 BKO* mice exhibited deficiencies in BAT development and thermogenic function, resulting in hypothermia with acute cold exposure. *Sox4 MKO* mice were easy to gain weight with HFD. Elevated expression of SOX4 in mice resulted in upregulation of thermogenic genes and increased heat production, thereby mitigating HFD-induced obesity. Besides, older or obese mice exhibited severely whitened BAT, and SOX4 expression in BAT was significantly lower. These results indicated that SOX4 is essential for BAT development and the maintenance of thermogenic function.

SOX4 is required for the early development of BAT during mouse embryonic stages. In *Sox4-MKO* mice, BAT depots diminished significantly compared to that in *Sox4*^*f/f*^ littermates, and immunostaining analysis revealed a notable decrease in UCP1 and EBF2 expression at E15.5. Additionally, SOX4 directly activates the transcription of EBF2, which is a key factor determining the cell fate of brown adipocytes. The scRNA-seq data from the dorsal-anterior region of mouse embryos at E10.5–E13.5 revealed that SOX4 and EBF2 exhibited a high degree of spatial coincidence in sclerotome cells as well as *Ebf2*^*+*^*/Cdh4*^*+*^ BAT preadipocytes, suggesting that SOX4 may play a crucial role in brown preadipocyte commitment. Furthermore, RNA-Seq data from human iPSCs differentiated into brown adipocytes revealed an initial observation of elevated levels of SOX4 on day 4 of differentiation, which is expressed as early as EN1 [39]. These suggest SOX4 plays a crucial role in embryonic BAT development.

SOX4 regulates BAT development and thermogenesis via EBF2. RNA-seq analysis revealed many downregulated thermogenic genes from *Sox4-MKO* were overlapped with down-regulated genes from *Ebf2 AKO* mice. Overexpression of EBF2 in SOX4 knockout SVF cells significantly rescued expression of thermogenic genes. Further, ChIP-qPCR results demonstrated that SOX4 binds to promoter of EBF2. SOX4 directly activated EBF2-luciferase in a manner dependent on its HMG and TAD domains. The HMG domain, which contains an L-shaped structure, binds to target DNA sequences and alters chromatin conformation to facilitate transcription [40, 41]. These findings suggested SOX4 may mediate remodeling of chromatin structure in activating EBF2 transcription. FAIRE-qPCR results revealed depletion of SOX4 significantly reduced chromatin accessibility of EBF2 promoter. EBF2, an essential transcriptional regulator of brown fat cell fate, can promote PPARγ to target to BAT-selective genes and thermogenic genes to maintain BAT identity [15]. Consistently, in BAT of *Sox4-MKO* mice, the binding of PPARγ to the *Prdm16* and *Ucp1* promoters decreased significantly. Additionally, the cooperative interaction between SOX4 and EBF2 leads to a robust transcriptional activation of downstream thermogenic genes. Therefore, SOX4 regulates brown fat formation and thermogenic function by mediating adipocyte remodeling via EBF2.

Besides, similar to *Ebf2*^*-/-*^ mice, SOX4-deficient mice exhibit the presence of adipocytes instead of a total lack of BAT. Other factors, such as Ebf1 or Ebf3, may compensate for the role of Ebf2 in adipocyte differentiation in *vivo* [15, 17]. Nevertheless, the brown characters and functions of BATs from SOX4-deficient mice are diminished. Loss of SOX4 results in lower expression EBF2, which recruits PPARγ to its brown-selective binding sites and reduces its binding to WAT-specific sites [15]. In the absence of SOX4/EBF2, PPARγ binding to WAT-specific sites is enhanced, resulting in a significant increase in the expression of WAT-selective genes that ultimately promote whitening of BAT. Additionally, genes associated with lipogenesis were upregulated, whereas those associated with lipolysis exhibited downregulation in BATs from *SOX4-MKO* mice, thereby facilitating triacylglycerol accumulation and contributing to whitening of BAT.

SOX4 plays a critical role in adaptive thermogenesis. Under acute cold exposure, SOX4 mRNA levels were increased both in BAT tissues and SVF cells. Due to defects in thermogenic gene program, SOX4-deficient mice had lower adaptive thermogenesis in response to acute cold exposure. In adult mice, *Sox4 MKO* and *Sox4 BKO* mice as well as *Sox4*^*f/f*^ mice had similar levels of heat production at room temperature. When mice were subjected to 4 °C, *Sox4*^*f/f*^ mice exhibited higher production of heat and consumption of O_2,_ while that of *Sox4 MKO* and *Sox4 BKO* mice was significantly lower. On normal chow, there was no significant difference in the heat production of *Sox4 MKO* and *Sox4*^*f/f*^ mice. When mice were fed with HFD, *Sox4 MKO* showed deficiency in thermogenesis and reduced O_2_ consumption. Due to decreased heat production and energy consumption, *Sox4 MKO* mice were prone to develop obesity and related metabolic diseases. Conversely, with SOX4 overexpressed in preadipocytes, *Pref1-Sox4* mice could produce more heat under acute cold exposure or HFD [23]. Overexpression of SOX4 with adenovirus or AAV promoted expression of thermogenic genes. In addition, we previously found that SOX4 is expressed in BAT as well as in WAT and SOX4 is required for beige adipocyte-mediated adaptive thermogenesis with prolonged cold exposure [23]. These findings suggest that enhancing the expression of SOX4 in adipocytes may represent a promising strategy for promoting energy expenditure to counteract obesity.

Notably, acute cold exposure treatment significantly upregulated the expression of SOX4 and EBF2, along with an increased proportion of BAT preadipocytes in BAT SVFs [35]. Cold stimuli can activate the cAMP-PKA pathway through adrenergic receptor signaling. Forskolin activates PKA, leading to the S235 phosphorylation of SOX4, which promotes its nuclear translocation and binding to the EBF2 promoter, thereby enhancing transcriptional activity of EBF2. These findings suggest that cold exposure may induce brown adipocyte formation by upregulating EBF2 expression mediated by PKA-phosphorylated SOX4.

There are still some issues that need to be addressed in further experiments. Our data from experiments with differentiated cells and mouse models have demonstrated that SOX4 is required for brown adipocytes differentiation. To further analyze the role of SOX4 in determining brown cell fate, lineage-tracing studies need to be confirmed in the future. Additionally, how the phosphorylation of SOX4 mediates its nuclear translocation and promotes its transcriptional activity remains unclear. S235A mutation of SOX4 failed to enter the nucleus and was not able to activate the transcription of EBF2 with forskolin treatment. Through the AlphaFold protein structure website (https://alphafold.ebi.ac.uk/), we analyzed the spatial localization of serine residues at position S235. It is located in the linker region between regular and irregular structures. Phosphorylation of S235 might alter the spatial conformation of SOX4, enabling it to be recognized by the nuclear pore protein complex for entry into the cell nucleus. Identifying factors that enable phosphorylated SOX4 to enter the nucleus may reveal new strategies to enhance thermogenesis and energy consumption.

Promoting BAT development and function might be a promising strategy to counteract obesity and related metabolic disorders [42, 43]. Short-term cold exposure enables brown fat remodeling in mouse and human [44, 45]. We have established that SOX4 is essential for brown fat development and maintenance. A notable decrease in SOX4 expression was observed in the whitened BATs of genetically modified or HFD-induced obese mice, as well as in aged mice. Our findings show that AAV-mediated overexpression of SOX4 enhances thermogenic capacity of BATs, helping mice resist HFD-induced obesity. Genetic modification of adipose tissue using AAV technology offers a promising strategy for developing innovative treatments for obesity-related metabolic disorders [46]. In addition, recent reports suggest that the application of human brown-like adipocytes in cell-based therapies presents significant therapeutic benefits in mouse models [47]. Elucidating the role and mechanisms by which SOX4 influences brown fat cell differentiation may facilitate the engineering of precursor cells into brown or beige adipocytes, potentially offering new avenues for future cellular therapies.

## Materials and methods

### Mice

Mice were housed in colony cages under specific conditions: temperature maintained at 22–24 °C, humidity at 50–60%, and a 12-hour light/dark cycle starting at 7:00 am. All mice were age-matched male mice with a C57BL/6 genetic background, and specific ages were detailed in the figure legends.

*Sox4*^*f/f*^ mice, generated by GemPharmatech Company (Nanjing, China), and *Pref1-Sox4* mice, generated by Cyagen Company (Guangzhou, China), were previously described [22, 23]. *Myf5-Cre* mice were provided by Prof. Wei Mo at Zhejiang University, and *Ucp1-Cre* mice were provided by Prof. Tongjin Zhao at Fudan University. *Sox4*^*f/f*^ mice were crossed with *Myf5-Cre* or *Ucp1-Cre* transgenic mice to generate *Sox4-MKO* or *Sox4-BKO* mice, respectively (Figs. 1A and  S1I). The *ob/ob* mice were purchased from Shanghai Model Organisms Center. The Taq MasterMix (CWBIO, Cat#CW0690H) was used for genotyping, and the genotyping primers are listed in Supplemental Table 1.

The chow diet (NCD, Xietong Organism, Nanjing, China) consists of 67.4% carbohydrates, 20.6% protein, 12% fat. The high-fat diet (HFD, Readydietech Co., Ltd., Shenzhen, P. R. China) contains 20% calories from carbohydrate, 20% calories from protein, and 60% calories from fat.

### Immunohistochemistry and immunofluorescence

For H&E staining, mice were euthanized, and indicated tissues or embryos were fixed in 4% paraformaldehyde (PFA) overnight at room temperature. The fixed tissues were dehydrated in ethanol, embedded in paraffin and sectioned at 4 μm. Sections were stained with hematoxylin and eosin (Wanleibio, Cat#WLA051a) according to manufacturer’s instructions.

For immunofluorescence, the slides were deparaffinized and performed heat-induced antigen retrieval with 10 mM sodium citrate. The slides were then blocked with 3% BSA and 0.1% Triton X-100 in PBS (Pricella, Cat#PB180327) for 1 hr at room temperature, followed by incubation with the indicated primary antibodies: SOX4 (Abcam, Cat#ab243041) (dilution 1:100), PRDM16 (Abcam, Cat#ab106410) (dilution 1:50), TOM20 (Santa Cruz, Cat#sc-17764) (dilution 1:100), EBF2 (Affinity, Cat#DF13398) (dilution 1:100), F4/80 (Abclonal, Cat#A18637) (dilution 1:100), Flag (Sigma-Aldrich, Cat#F7425) (dilution 1:200), UCP1 (Abclonal, Cat#A5857) (dilution 1:100). Subsequently, the slides were washed three times with PBS and incubated with indicated secondary fluorescent-conjugated antibodies: Alexa Fluor 488 (Invitrogen, Cat#A11029) (dilution 1:1000), Alexa Fluor Plus 647 (Invitrogen, A32733) (dilution 1:2000), at room temperature for 1 h in the dark. Finally, the slides were washed three times with PBS for 5 minutes and incubated with 5 μg/μl DAPI (Mei5 Biotech) for 10 minutes. For mitochondrial quantification of BAT tissue, the mitochondrial marker Tom20 was selected for mitochondrial quantity analysis. Samples were observed using the Zeiss LSM 780 confocal microscope or Leica Aperio Versa 200 microscope [48].

For immunohistochemistry, paraffin-embedded tissues underwent deparaffinization, rehydration, and antigen retrieval. The sections were then treated with 3% H_2_O_2_, permeabilized with 0.1% Triton X-100, and incubated with the UCP1 antibody overnight at 4 °C. Subsequently, adipocytes labeled with the UCP1 antibody were visualized using goat anti-rabbit IgG conjugated with HRP (EpiZyme, Cat#LF101) (dilution 1:5000), following the instructions of the DAB chromogenesis kit (Elabscience, Cat#E-IR-R217).

### Transmission electron microscopy

Dissected BATs were cut into small pieces and fixed overnight at 4 °C in pre-cooled fixation buffer (2.5% glutaraldehyde, 0.1 M phosphate buffer, pH 7.4). The fixed samples were rinsed three times with 0.1 M phosphate buffer and post-fixed in 1% OsO_4_ for 2 hr. Then the samples were rinsed and dehydrated sequentially in concentration gradient ethanol. After embedding and slicing, thin sections were stained with uranyl acetate and lead citrate before imaging. Electron microscopy (Hitachi, HT-7800) was utilized for image capture [49].

### Body-composition analysis

The mice were immobilized with the indicated tool and then positioned in the instrument for body composition analysis. The Echo MRI composition analyzer (Echo Medical Systems, 100H) was employed to assess the fat mass and lean mass data of the mice.

### Infrared thermography and cold tolerance test (CTT)

Mice were genotyped on postnatal day 1. The 1-day-old male mice were housed individually for 30 minutes at room temperature, and the surface temperature was immediately obtained with an infrared imaging device (Junctec, Ax5).

For CTT, the mice were individually housed in cages at 22 °C for one week prior to the test. On the day of experiment, mice were fasted for 4 h with water and then moved to individual cages at 4 °C. Rectal temperature was measured with a rectal thermocouple probe (NJKEWBIO, FT3400) at indicated time. At the end of these experiments, the mice were euthanized for subsequent experiments [50].

### Glucose and insulin tolerance tests

For glucose tolerance test, the mice were fasted for 16 hr with assess to water only, and then they received an intraperitoneal injection of D-glucose at a dose of 1.0 g/kg body weight. For insulin tolerance test, mice were starved for 6 hr with assess to water and then intraperitoneally injected with human insulin at a dose of 1.5 U/kg. Blood samples were collected from the tail vein at 0, 15, 30, 60, 90 and 120 minutes after the intraperitoneal injection, and blood glucose levels were measured using a glucometer.

### Metabolic cage study

Before this experiment, the mice were individually housed at 22 °C for a week and then acclimatized to the metabolic cages for 2 days. The Sable Promethion system was employed to meticulously record various parameters including food intake, body weight, oxygen consumption, heat production, and locomotor activities.

To evaluate metabolic data during acute cold challenge, the instrument temperature was adjusted to 4 °C after fasting the mice for 4 hr. Oxygen consumption and heat production were continuously monitored during this period.

To test metabolic data after intraperitoneal injection of CL316,243 (MCE, Cat#HY-116771A), *Sox4*^*f/f*^ and *Sox4-MKO* mice were injected with CL316,243 (1 mg/kg) intraperitoneally. The mice were then moved to metabolic cage, and oxygen consumption and heat production were monitored.

### Western blot analysis

The protein from tissues or cells were lysed using RIPA lysis buffer (APExBIO, Cat#K1120, Houston, USA) supplemented with phosphatase inhibitor and proteinase inhibitor cocktail (TargetMol, Cat#C0001). After ultrasonication, the lysates were centrifuged at 13,000 g for 10 min at 4 °C. The homogenized supernatants were quantified using a BCA protein assay kit (NCM biotech, Cat#WB6501). The protein samples were separated on SDS-PAGE (CYTOCH, Cat#PW0002) and transferred onto hydrophobic PVDF membrane (Millipore, Cat#IPVH00010). The membranes were blocked with 5% defatted milk powder at room temperature for 1 hr and then incubated with indicated primary antibodies, including SOX4 (Abcam, Cat#ab70598) (dilution 1:200), PCG1a (Millipore, Cat#AB3242) (dilution 1:1000), AGT (Abclonal, Cat#A11689) (dilution 1:1000), OXPHOS (Abcam, Cat#ab110413) (dilution 1:1000), Flag (Sigma-Aldrich, Cat#F7425) (dilution 1:1000), HA (Bioss, Cat#BMS0966M) (dilution 1:1000), PPARγ (Bioworld, Cat#BS79617) (dilution 1:1000), β-actin (Sino Biological, Cat#109444-T36) (dilution 1:10000), UCP1 (Diagbio, Cat#db9840) (dilution 1:1000), PRDM16 (Abcam, Cat#ab106410) (dilution 1:500), PLIN1 (Boster, Cat#AAED-16) (dilution 1:1000), HSL (ZENBIO, Cat#344379) (dilution 1:1000), p-HSL-S660 (SAB, Cat#12416) (dilution 1:1000), p-PKA Substrate (CST, Cat#9621) (dilution 1:1000). Subsequently, the membranes were incubated with HRP-conjugated secondary antibodies: Goat Anti-Rabbit IgG Antibody (GenScript, Cat#A00098) (dilution 1:5000), Goat Anti-Mouse IgG Antibody (GenScript, Cat#A00160) (dilution 1:5000). The membrane was visualized with enhanced chemiluminescence detection (Abbkine, Cat#BMU101).

### Gene expression analysis

Total RNA was extracted from tissues or cells using TRIzol reagent (Accurate Biology, Cat#AG21101) according to standard protocols and dissolved in DEPC H_2_O. cDNA was synthesized by cDNA synthesis kit (Abm, Cat#G490), and quantitative PCR (qPCR) was performed with a real-time PCR system (Bio-Rad) using SYBR Green (Swiss Affinibody LifeScience AG, Cat#Q01). *18S* mRNA was used as the invariant control. The primers sequences are listed in Supplementary Table 1.

For RNA-Seq, BAT tissues were isolated from 15-week-old male *Sox4-MKO* mice and control littermate mice (n = 2). The total RNA was extracted using TRIzol Reagent, and 4 µg of total RNA was used to construct biologically sequencing libraries essentially according to Illumina TruSeq RNA Sample Preparation v2 Guide. The samples were amplified by PCR and sequenced using Illumina HiSeq2500 by AMOGENE (Xiamen, China). Genes with *p*-value < 0.05 and averaged FPKM value > 1 in at least one genotype were defined as *Sox4*-regulated genes. Heat maps and clustering analysis were conducted by the GENE DENOVO platform available at https://www.omicshare.com/tools/Home/Soft/heatmap.

### Cell culture and adipocyte differentiation

HEK293T (ATCC, Cat#CRL-3216) and NIH3T3 (Pricella, Cat#CL-0171) cells: Cells were cultured in growth media containing high-glucose DMEM (Viva Cell, Cat#C3103-0500), 100 U/ml penicillin, 100 mg/ml streptomycin (Biochannel, Cat#BCCE007), 1 mM sodium pyruvate (BasalMedia, Cat#S410JV), 1% NEAA (IMMOCELL, Cat#IMC-D07) and 10% FBS (EXCell Bio, Cat#FSP500) at 37 °C in an atmosphere of 5% CO_2_.

For the immortalization of BAT preadipocytes, postnatal day 1 pups of wild-type C57BL/6 mice were sacrificed, and BAT tissue was collected. Brown preadipocytes were isolated using 3 mg/mL collagenase II (Sigma-Aldrich, Cat#C6885) at 37 °C for 45 minutes, following a previously described method [51]. The SVFs were cultivated in high-glucose DMEM (Sunncell) supplemented with 20% FBS (CellMax, Cat#SA111) at 37 °C in an atmosphere of 8.8% CO_2_ and treated with a large T-antigen retrovirus for 2 days. Subsequently, they were cultured in fresh medium containing G418 (Aladdin, Cat#108321-42-2) (50 μg/ml) for 4 days to select immortalized cells.

For mature brown adipocytes differentiation, the immortalized cells were cultured until they reached 95% confluence and then treated with Medium A containing 1 nM T3 (Sigma-Aldrich, Cat#T2877), 0.125 mM indomethacin (Sigma-Aldrich, Cat#I7378), 1 μM rosiglitazone (MCE, Cat#HY-17386), 5 μM dexamethasone (Sigma-Aldrich, Cat#D1756), 850 nM insulin (MCE, Cat#HY- P0035), and 0.5 mM IBMX (Sigma-Aldrich, Cat#I5879) for 2 days. After 48 hours, cells were treated with Medium B containing 1 nM T3, 1 μM rosiglitazone and 850 nM insulin. Starting from the fourth day, the cells were cultured in medium B, and the medium was changed every two days until harvest. On day 6, mature brown adipocytes were achieved and harvested for indicated assay.

### Oil Red O staining and triglyceride measurement assay

For cell staining, a 0.35% (w/v) Oil Red O stock solution (Solarbio, Cat#G1262) was prepared beforehand. The working solution was created by mixing three parts of the stock solution with two parts of water. The cells were rinsed with PBS and then fixed with 4% formalin at room temperature for 15 minutes. After fixation, the cells were incubated with the Oil Red O working solution for 10 minutes at room temperature in cell culture dish (Jet Biofil). Then, the samples were washed three times and subjected to microscopic. For triglyceride levels measurement, the mature brown adipocytes were suspended in PBS and fragmented on ice using an ultrasonic processor (Cole Parmer) at 20% amplitude. The resulting cell lysate was used for triglyceride measurement, and the data were normalized to the protein concentration in the lysate.

### Oxygen consumption rate (OCR) measurements of brown adipocytes

BAT SVF cells were plated into XFe96 cell culture microplate (Agilent) and differentiated into brown adipocytes, followed by OCR measurement using an XF96 Extracellular Flux Analyzer (Seahorse Bioscience) according to the manufacturer’s instructions. Before OCR measurement, the cell culture medium was replaced with Seahorse XF DMEM (Agilent, Cat#103575-100) supplemented with 25 mM glucose, 1 mM sodium pyruvate, and 2 mM glutamine (Keygen BioTECH). During OCR measurement, 4 µM oligomycin, 2.5 µM FCCP and 1.5 µM rotenone/antimycin were sequentially injected into the microplate to detect the uncoupled respiration, maximal respiration and non-mitochondrial respiration, respectively. OCR was normalized to the protein content.

### Serum analysis

Before the mice were sacrificed, the blood was obtained from the eyeballs and clotted at 4 °C for 4-6 hr. The samples were then centrifuged at 1500 g, 4 °C for 10 minutes and the supernatants were collected. The commercial assay kits were utilized for TG (Nanjing Jiancheng, Cat#A110-1-1) and FFA (Bioswamp, Cat#BTK026) measurements, following the manufacturer’s instructions.

### Plasmids, lentivirus packaging and infection

For the construction of lentiviral overexpression plasmids, mouse *Sox4* and *Ebf2* were cloned from a mouse cDNA library obtained from mouse BAT tissue. The genes were inserted into either pLV-N-Flag/HA-XM vector (provided by Dr. Jiahuai Han at Xiamen University) or pCDH-EF1-MCS-IRES-BSD vector (provided by Dr. Tongjin Zhao at Fudan University) with indicated tags by Seamless Assembly Kit (Shanghai Acmec Biochemical Technology Co., Ltd, Cat# AC17180). For knockdown, indicated shRNA were cloned into pLKO.1 plasmid (Addgene). The constructed plasmids were extracted according to the instructions of the plasmid extraction kit (Shandong Sparkjade Biotechnology, Cat#AD0103). For lentivirus production, the constructed lentivirus plasmids were transfected into HEK293T cells along with pHR and pVSV-G plasmids, following previously described protocols [52]. After 48 hr, the lentivirus were collected and concentrated (Sartorius, Cat#VS15T42). For lentivirus infections, cells were cultured to 75% confluence and then infected with lentiviruses in medium supplemented with 10 μg/ml polybrene (HUAYUN, Cat#HYP490). After 36 hr, the infected cells were seeded into new dished and selected with 5 μg/ml puromycin (GIBCO, Cat#11138-03) for 1 week to establish stable cell line. The primer sequences are listed in Supplementary Table 1.

### Mitochondria quantity analysis

BATs were isolated and finely minced into small pieces with scissors. The tissue was then digested overnight at 55 °C in TNES digestion buffer (0.2 M NaCl, 0.1 M Tris, 5 mM EDTA, 0.4% SDS) supplemented with 200 μg/ml protease K (Macklin, Cat#P6321). Following digestion, genomic DNA was extracted using 6 M NaCl, and subsequently precipitated with 100% ethanol. After drying, the DNA was resuspended in ddH_2_O. For mitochondria quantity analysis, specific coding genes for NADH dehydrogenase 1, 2, or 4 (*Nd1*, *Nd2*, or *Nd4*) were selected, and the cyclophilin (*Ppib*) gene was utilized as a reference. The sample were determined by RT-PCR, the primer sequences are listed in Supplementary Table 1.

### Construction, purification, and injection of adenovirus

For the construction of the adenoviral vector, the CDS region of *Sox4* gene was cloned into a shuttle vector pAdTrack-CMV to form pAdTrack-CMV-Sox4 plasmid by T4 DNA ligase (CUSABIO, Cat#CSB-YP355583EDZ). The pAdTrack-CMV-Sox4 plasmid was then linearized by digestion with PmeI (Novoprotein, Cat#RE106-U050) and co-transfected into Escherichia coli strain BJ5183 along with an adenoviral backbone plasmid called pAdEasy-1. The recombinant clones were selected based on their resistance to kanamycin (YuanYe, Cat#Y69596) and verified by restriction endonuclease analysis. Subsequently, the recombinant plasmid was extracted and linearized using Pac I restriction enzyme. The linearized recombinant plasmid was then transfected into β-5 cells cultured in 15 cm dishes (NEST Biotechnology) for packaging into adenovirus particles.

For purification of the adenovirus, cells and supernatants were collected when approximately one-third to half of the cells were detached. The collected samples were subjected to three cycles of freeze-thawing to release the viral particles. Next, the precipitated viral particles were purified through PEG8000 (Biofroxx, Cat#1363GR500) precipitation. The resulting precipitates were further purified using cesium chloride density gradient centrifugation at 22800 g and 20 °C for 2.5 hr. The adenovirus bands were carefully collected by puncturing the centrifuge tubes and dialyzed overnight. The titer of the purified adenovirus was determined, and the purified adenovirus was subpackaged for future use.

To achieve overexpression of SOX4 in BAT of *Sox4-MKO* mice, adenoviruses carrying the SOX4 were injected into BAT pads within a designed area at 10^10^ *pfu* per mice. The adenoviruses were injected twice every 3 days. At day 7, mice were euthanized, and BAT tissues from the injected area were collected for indicated assays.

### Packaging and purification of adeno-associated virus (AAV)

In the study, the AAV2/9 system was utilized for overexpression of SOX4 in BAT. To construct the adeno-associated virus plasmid, the CDS region of *Sox4* gene was cloned into pAAV-mini-*Ucp1*-GFP vector, generously provided by Dr. Shengcai Lin at Xiamen University. Then the transgene plasmid was transfected into HEK293T cells along with packaging plasmids AAV2/9 and ΔT-6 by transfection reagent (AbBOX, Cat#KX0110055). After 72 hr of transfection, both the cells and the medium containing the packaged viruses were collected. The cells were lysed through three rounds of freeze-thawing to release the viral particles. The supernatants, along with the culture medium containing viruses, were collected and precipitated using 5 × polyethylene glycol (PEG) at a final concentration of 8% PEG-8000 and 0.5 M NaCl. Subsequently, the precipitates were purified through iodixanol (Sigma-Aldrich, Cat#92339-11-2) density gradient ultracentrifugation (17%, 25%, 40%, and 60%). qPCR with specific primers was employed to determine the copy numbers of AAV. For in vivo experiments, AAVs carrying SOX4 constructs were injected into the tail vein of 6-week-old male mice at a dosage of 0.4×10^12^ copies per mouse, and littermates were injected with equal amount of AAV carrying the empty vector as the control group. After a period of 5 weeks, the mice were euthanized, and the effectiveness of AAV-mediated gene modulation was examined. The primer sequences can be found in Supplementary Table 1.

### Luciferase reporter assays

To construct the luciferase reporter, we amplified the promoter region of the specified genes from mouse genomic DNA and inserted it into the pGL3-basic or pGL4.26 plasmid (Promega). Cells were cultured until they reached approximately 75% confluence. Subsequently, the constructed luciferase reporter plasmid, along with β-galactosidase (β-gal) and other specified plasmids, were transfected into the cells using EZ-Trans transfection reagent (Shanghai Life-iLab Biotech, Cat#AC04L092). After a period of 36-48 hr post-transfection, the cells were harvested and subjected to luciferase and β-galactosidase assays. The activity of luciferase serves as an indicator of gene expression, and β-galactosidase is used as an internal control. The luciferase activity was then normalized to the corresponding β-galactosidase levels to address any variations and ensure accurate calibration of experimental errors.

### Chromatin immunoprecipitation (ChIP) and ChIP-sequencing analysis

The immortalized BAT SVF cells were infected with lentiviruses expressing either pLV-Flag-empty or pLV-Flag-Sox4 constructs, and the infection was allowed to proceed for 48 hours, establishing a stable cell line. Subsequently, the cells were cultured until they reached 100% confluence and were then treated with DMSO or forskolin (MCE, Cat#HY-15371) (20 μM) for 1 hr before being harvested. For fixation, the cells were treated with 1% PFA at 37 °C for 15 minutes in 100 mm cell culture dish (SAINING). To stop the fixation process, 0.125 M glycine was added and incubated for 5 minutes at room temperature. Following fixation, the cells were washed with cold PBS supplemented with 1 mM PMSF and lysed with ChIP SDS lysis buffer (50 mM Tris-HCl, pH 8.1, 10 mM EDTA, 1% SDS) containing protease inhibitor and phosphatase inhibitors for 15 minutes on ice. To achieve DNA fragmentation within the desired range of 200-800 base pairs, an ultrasonic processor (SCIENTZ, Cat#SCIENTZ08-IIIA) was used on ice. Subsequent steps were performed according to the specific biotechnology protocol, and the enrichment of DNA was measured using qPCR and normalized with the input sample. The primer sequences can be found in Supplementary Table 1.

To investigate the genomic binding patterns of SOX4, we established a stable cell line of BAT SVF overexpressing 3X HA-SOX4 for ChIP-Seq assays. ChIP-Seq assay was performed as above, except purified DNA was used for library construction and sequencing.

The library was prepared utilizing a KAPA Hyper Prep Kit as per the provided guidelines. Adaptors and primer sequences from Roche were employed for both library construction and amplification. Subsequent to PCR amplification, fragments ranging from 250 to 450 bp were isolated by employing KAPA pure beads as per the manufacturer’s instructions. All ChIP libraries underwent pair-end sequencing on HiSeq3000 systems at Sangon Biotech (Shanghai, China).

### Immunoprecipitation

The cells were lysed with IP buffer (MKbio, Cat#MP1504) supplemented with protease inhibitors and phosphatase inhibitors. The total cell lysates were incubated with indicated antibodies or FLAG Magnetic Beads (HUABIO, Cat#HAK21011) at 4 °C for 8 hr. Then protein A/G magnetic beads (MCE, Cat#HY-K0202) were washed with IP buffer for 3 times, and the above cell lysate were incubated with beads. The eluted fraction was analyzed by western blot using indicated antibodies.

### Formaldehyde-assisted isolation of regulatory elements (FAIRE) assay

The cells were cultured to 95% confluence and then fixed in 1% PFA 15 minutes at room temperature. The cross-linking reaction was quenched by adding 0.125 M glycine (LABLEAD, Cat#GAS10-1) for 5 minutes at room temperature. After washing with cold PBS supplemented with 1 mM PMSF, the samples were collected, the cells were lysed in SDS lysis buffer (1% SDS, 10 mM EDTA, 50 mM Tris-HCl, pH 8.1, 1 mM PMSF and 1 mM protease inhibitor cocktail) for 10 minutes on ice. To obtain DNA fragments ranging from 200 to 1,000 bp, the cell lysates were sonicated under appropriate conditions. After centrifugation, the supernatant from each group was collected and incubated with RNase A (YEASEN, Cat#10405ES03) at 37 °C for 1 hr. Subsequently, the samples were divided into two equal parts. For de-crosslinking (control DNA), 10 µL of proteinase K (20 mg/mL) was added and incubated at 37 °C for 4 hr, followed by incubation at 65 °C for 6 hr. The non-de-crosslinked and de-crosslinked samples were then purified using the phenol:chloroform method. Chromatin accessibility was assessed using qPCR and analyzed according to a previously reported calculation method. The primers used for FAIRE-PCR were listed in Supplementary Table 1.

### scRNA-seq analysis and spatial deconvolution analysis

For the scRNA-seq analysis of mouse embryos ranging from E10.5 to E13.5, we utilized a publicly available dataset (GSE233955) and conducted the analysis using the R package Seurat v4. Quality control involved setting cutoffs for the maximum percentage of reads mapping to mitochondrial genes for each dataset separately. Scrublet was employed to remove potential doublets. Subsequently, we regressed out cell cycle phase, percentage of reads mapping to mitochondrial genes, and percentage of reads mapping to histone genes using the scale data function. After performing principal component analysis (PCA), we reduced the dimensionality of each dataset using uniform manifold approximation and projection (UMAP) implemented in the RunUMAP function. Clusters were defined using the find neighbors and find clusters functions. Fibroblasts and skeletal muscle cell clusters were identified based on the expression of *Pdgfrα* and *Tnnt1*, respectively, at each embryonic stage. We then used the top 3000 variable genes to create potential anchors with the find integration anchors function and integrated the data using the integrate data function, resulting in a new matrix with 3000 features. Subsequently, we selected cell clusters predicted to localize to the dorsal mouse embryo by patial deconvolution analysis. Next, we conducted cell type deconvolution based on spatial transcriptomic data using SpaDecon. We obtained spatial transcriptomics datasets from sagittal sections of E10.5-E13.5 mouse embryos from the mouse organogenesis spatial transcriptomics atlas (MOSTA). The scRNA-seq dataset spanning E10.5-E13.5 was used to deconvolve the MOSTA data corresponding to E10.5, E11.5, E12.5, and E13.5. The indicated code details are available at https://github.com/luoy-cloud/Sox4-program/tree/main.

### Quantification and statistical analysis

All data in this study were presented as mean ± SEM and analyzed using GraphPad Prism 8.0 software. Each group of data was analyzed for normal distribution using the Anderson-Darling test, D’Agostino-Pearson test, Shapiro-Wilk test, or Kolmogorov-Smirnov test, as appropriate. An unpaired two-tailed Student’s *t*-test was used to determine significance between two groups of normally distributed data. An unpaired two-tailed Mann–Whitney test was used to determine significance between data without a normal distribution. Multiple group comparisons were performed using the Kruskal-Wallis test followed by Dunn’s multiple comparisons test, or one-way or two-way ANOVA followed by Tukey’s test, as indicated in the legends. Immunoblotting assay analysis and lipid droplet size analysis were performed using ImageJ software (National Institutes of Health). The statistical details of experiments were prepared and given in the figure legends, including exact number of mice samples and data analysis methods. Except for high-throughput sequencing experiments, at least three biological replicates were performed for mouse experiment and cell experiment. Significant differences of all results are indicated as *p < 0.05, ** *p* < 0.01, and *** *p* < 0.001, ns, no significance. Investigators were not blinded to group allocation during experiments.

## Supplementary information

Supplementary information

Supplemental table 1

Uncropped western blots

## Data Availability

The datasets in this study are available in the following datasets: RNA-Seq data: Gene Expression Omnibus GSE263445. ChIP-Seq data: Gene Expression Omnibus GSE263446. All data needed to evaluate the conclusions in the paper are present in the paper and/or the Supplementary Materials. Additional data related to this paper may be requested from the authors.
