# Supplementary material for: SOX4 facilitates brown fat development and maintenance through EBF2-mediated thermogenic gene program in mice
Source: Cell Death Differ. 2024 Oct 15;32(3):447–65. doi: 10.1038/s41418-024-01397-0 (PMC11893884; doi:10.1038/s41418-024-01397-0)
Supplement: Supplementary file 1 — Supplementary information [file 41418_2024_1397_MOESM1_ESM.pdf]

Supplementary Materials for

SOX4 facilitates brown fat development and maintenance through EBF2-mediated thermogenic gene program in mice

Shuai Wang[1, 2, 5], Ting He[1, 5], Ya Luo[3, 5], Kexin Ren[1], Huanming Shen[1,4], Lingfeng Hou[1], Yixin Wei[1], Tong Fu[1], Wenlong Xie[1], Peng Wang[1], Jie Hu[1], Yu Zhu[1], Zhengrong Huang[2], Qiyuan Li[3, *], Weihua Li[2, *], Huiling Guo[1, *], and Boan Li[1, 6, *]

Supplementary figures：

Fig.S1. SOX4 is required for BAT development.

Fig.S2. There are no apparent defects of muscle in *Sox4-MKO* mice.

Fig.S3. Overexpression of SOX4 promotes thermogenic function of BAT.

Fig.S4. *Sox4-MKO* mice do not develop obesity on normal chow.

Fig.S5. Overexpression of SOX4 in BAT attenuates HFD-induced obesity.

Fig.S6. Loss of SOX4 downregulates lipolysis genes and upregulates lipogenesis genes.

Fig.S7. SOX4 is required for BAT-selective thermogenic genes expression.

Fig.S8. SOX4 is required for brown adipocytes differentiation *in vitro*.

Fig.S9. SOX4 is required for transcription of EBF2 in brown adipocyte differentiation.

Fig.S10. SOX4 cooperates with EBF2 to activate the transcription of Ebf2.

Fig.S11. SOX4 cooperates with EBF2 to promote transcription of thermogenic genes.

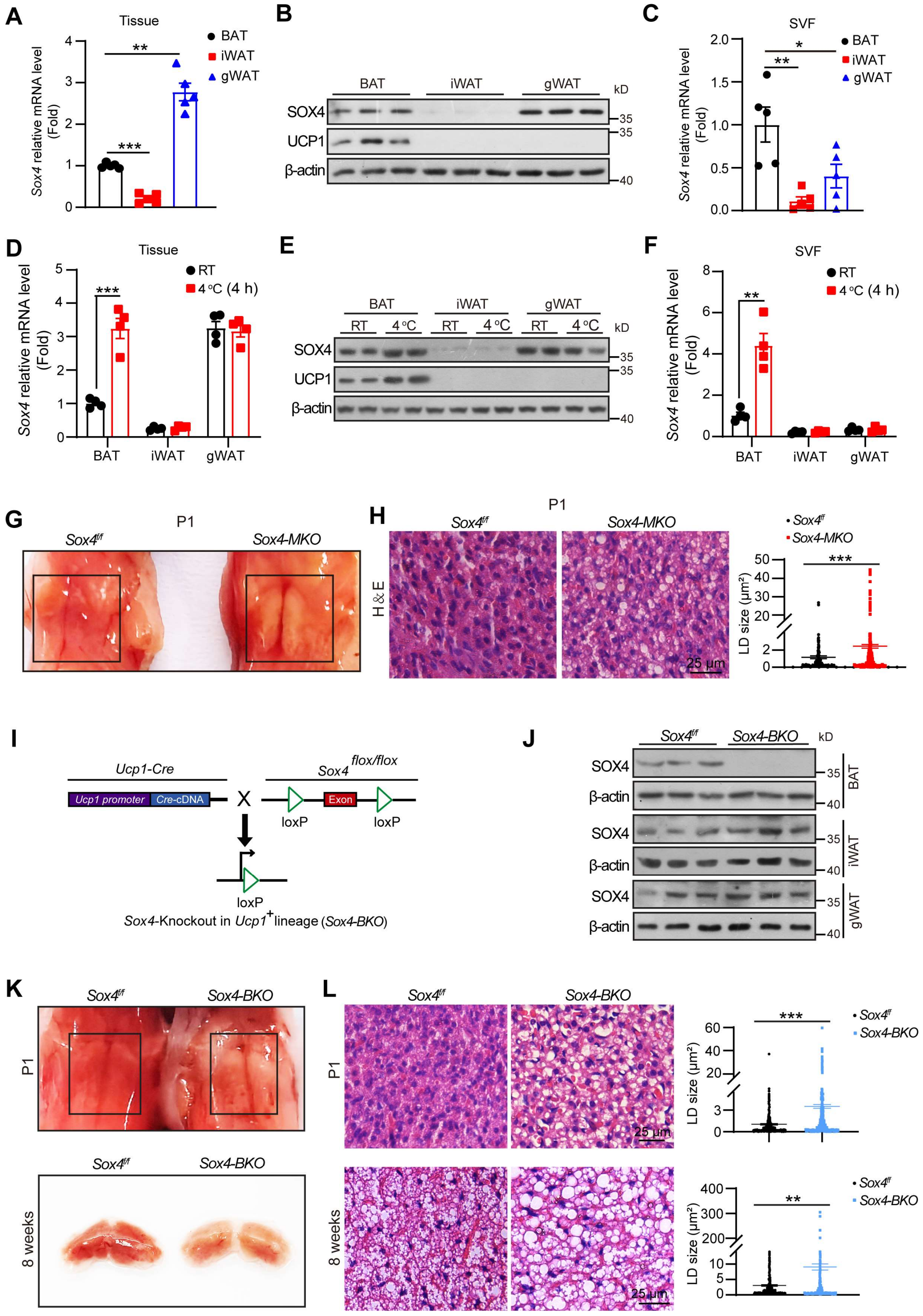
A
Tissue
BAT
iWAT
gWAT
Sox4 relative mRNA level (Fold)
B
SOX4
UCP1
β-actin
kD
C
SVF
D
RT
4 °C (4 h)
E
F
G
P1
Sox4f/f
Sox4-MKO
H
H & E
LD size (μm²)
25 μm
I
Ucp1-Cre
Ucp1 promoter
Cre-cDNA
Sox4 flox/flox
Exon
loxP
Sox4-Knockout in Ucp1+ lineage (Sox4-BKO)
J
Sox4-BKO
K
8 weeks
L

Fig.S1. SOX4 is required for BAT development. (Related to Fig.1)

A, **B** The mRNA levels (**A**) and protein levels (**B**) of SOX4 and UCP1 in BAT, iWAT and gWAT from 8-week-old male mice (n = 5). *18S* was used as an invariant control. The mRNA levels in BAT tissue were normalized to 1.0. **C** The mRNA levels of SOX4 in BAT SVF, iWAT SVF and gWAT SVF from 8-week-old male mice (n = 5). *18S* was used as an invariant control. The mRNA levels in BAT SVF were normalized to 1.0. **D**, **E** The mRNA levels (**D**) and protein levels (**E**) of SOX4 in BAT, iWAT and gWAT from WT mice (8-week-old male, n = 4) housed at room temperature (RT) or 4 °C for 4 h. *18S* was used as an invariant control. The mRNA levels in BAT tissue (RT) were normalized to 1.0. **F** BAT SVFs, iWAT SVFs and gWAT SVFs were isolated from WT mice (8-week-old male, n = 5) housed at RT or 4 °C for 4 h, then the mRNA levels of SOX4 were analyzed by qPCR. *18S* was used as an invariant control. The mRNA levels in BAT SVF (RT) were normalized to 1.0. **G**, **H** Photographs (**G**) and H&E staining of interscapular BATs (**H,** left) from 1-day-old $Sox4^{f/f}$ and *Sox4-MKO* male mice. The lipid droplets (LDs) sizes were quantified by ImageJ (**H**, right). Scale bar, 25 μm. **I** The strategy of generating *Sox4-BKO* is achieved by intercrossing *Ucp1-Cre* mice with $Sox4^{f/f}$ mice. **J** The protein levels of SOX4 in BAT, iWAT and gWAT of 8-week-old *Sox4-BKO* mice and control mice. **K**, **L** Images of BATs from $Sox4^{f/f}$ and *Sox4-BKO* male mice (P1, top panel; 8-week-old, bottom panel) (**K**). Representative H&E staining images of BATs from $Sox4^{f/f}$ and *Sox4-BKO* male mice (**L**, left). The LDs sizes were quantified by ImageJ (**L**, right). Scale bar, 25 μm. Asterisks (*) denote the level of statistical significance. $^{*}p < 0.05$, $^{**}p < 0.01$, $^{***}p < 0.001$. Data are presented as mean ± SEM. Statistical analyses were determined by unpaired two-tailed

Student's *t*-test (**D**, **F**), unpaired two-tailed Mann-Whitney test (**H**, right; **L**, right), and one-way ANOVA followed by Tukey's test (**A**, **C**).

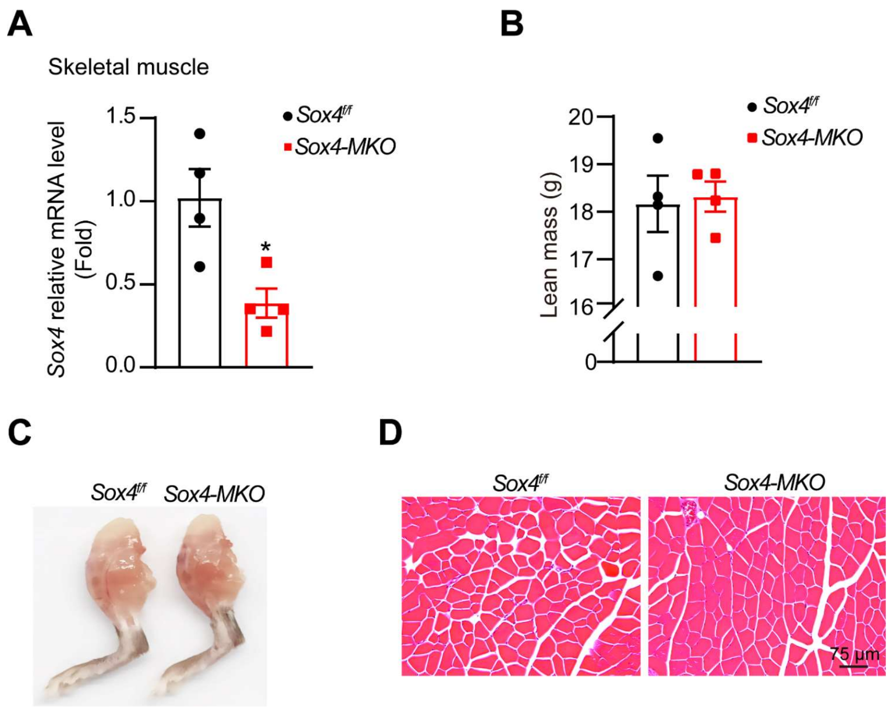

Fig.S2. There are no apparent defects of muscle in *Sox4-MKO* mice. (Related to Fig.2)

A The mRNA levels of SOX4 in skeletal muscle from 8-week-old male mice (n = 4). *18S* was used as an invariant control. The mRNA levels in control mice were normalized to 1.0. **B** The average lean masses of *Sox4*$^{f/f}$ and *Sox4-MKO* male mice (9-week-old, n = 4). **C** Photographs of quadriceps of *Sox4*$^{f/f}$ and *Sox4-MKO* male mice at 9-weeks old of age. **D** H&E staining of skeletal muscle from *Sox4*$^{f/f}$ and *Sox4-MKO* male mice at 9-weeks old of age. Scale bar, 75 μm. Asterisks (*) denote the level of statistical significance. *$p < 0.05$. Data are presented as mean ± SEM. Statistical analyses were determined by unpaired two-tailed Student's *t*-test (**A**, **B**).

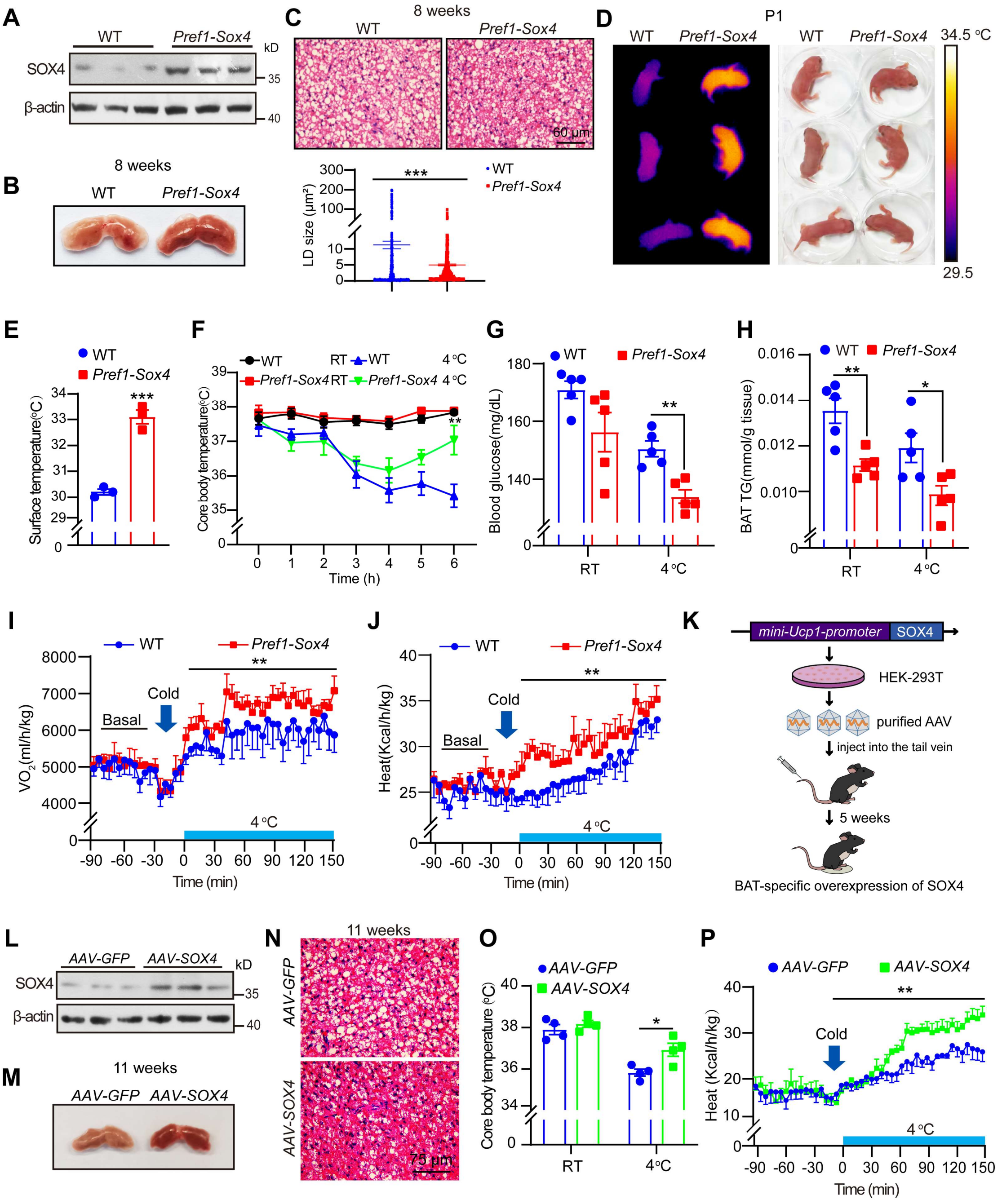
A
WT
Pref1-Sox4
kD
SOX4
35
β-actin
40
B
8 weeks
WT
Pref1-Sox4
C
8 weeks
WT
Pref1-Sox4
60 μm
LD size (μm²)
***
WT
Pref1-Sox4
D
P1
WT
Pref1-Sox4
WT
Pref1-Sox4
34.5 °C
29.5
E
WT
Pref1-Sox4
Surface temperature(°C)
***
F
WT RT
WT 4 °C
Pref1-Sox4 RT
Pref1-Sox4 4 °C
Core body temperature(°C)
Time (h)
G
WT
Pref1-Sox4
Blood glucose(mg/dL)
RT
4 °C
H
WT
Pref1-Sox4
BAT TG(mmol/g tissue)
RT
4 °C
I
WT
Pref1-Sox4
VO2(ml/h/kg)
Basal
Cold
4 °C
Time (min)
J
WT
Pref1-Sox4
Heat(Kcal/h/kg)
Basal
Cold
4 °C
Time (min)
K
mini-Ucp1-promoter
SOX4
HEK-293T
purified AAV
inject into the tail vein
5 weeks
BAT-specific overexpression of SOX4
L
AAV-GFP
AAV-SOX4
kD
SOX4
35
β-actin
40
M
11 weeks
AAV-GFP
AAV-SOX4
N
11 weeks
AAV-GFP
AAV-SOX4
75 μm
O
AAV-GFP
AAV-SOX4
Core body temperature (°C)
RT
4°C
P
AAV-GFP
AAV-SOX4
Heat (Kcal/h/kg)
Cold
4 °C
Time (min)

Fig.S3. Overexpression of SOX4 promotes thermogenic function of BAT. (Related to Fig. 2)

A The protein levels of SOX4 in BAT SVF cells isolated from *Pref1-Sox4* mice and its control mice (9-week-old male mice, n = 3). **B** Images of BATs from 8-week-old *Pref1-Sox4* mice and control male mice. **C** Representative H&E staining of BATs (top panel) from 8-week-old *Pref1-Sox4* mice and control male mice, and the LDs sizes were quantified and shown in bottom panel. Scale bar, 60 μm. **D**, **E** Infrared imaging of pups (postnatal day 1) of *Pref1-Sox4* mice and their control mice (**D**), and skin temperatures from the infrared images were quantified and shown in (**E**) (n = 3). **F**-**H** The changes in core body temperature of 9-week-old WT and *Pref1-Sox4* male mice at room temperature (n = 5) or under acute cold challenge at 4 °C for 6 h (n = 5) (**F**). At the end of the experiment, mice were sacrificed, and the blood glucose (**G**) and triglyceride content of BAT (**H**) were measured (n = 5). **I**, **J** *Pref1-Sox4* mice and control male mice (9-weeks-old) were switched from 22 °C to 4 °C, and the oxygen consumption (**I**) and heat production (**J**) were monitored by metabolic cage for 150 min (n = 5). **K.** Design of the assay for generating a mouse model with BAT-specific expression of SOX4. The transgene plasmids, which carried the mini-promoter of *Ucp1* for BAT-specific expression of SOX4, were transfected into HEK293T cells along with the indicated helper plasmids. The viruses were extracted and injected into 6-week-old male mice via the tail vein. After five weeks, the mice were utilized in the experiments. **L** The protein levels of SOX4 in BAT of *AAV-GFP* and *AAV-SOX4* mice. **M**, **N** The images (**M**) and H&E staining (**N**) of the BAT from *AAV-GFP* and *AAV-SOX4* mice. Scale bar, 75 μm. **O** The core rectal temperature of *AAV-GFP* and *AAV-SOX4* mice

recorded at RT or cold exposure for 4 h (n = 4). **P** *AAV-GFP* and *AAV-SOX4* mice were switched from 22 °C to 4 °C, and heat production were monitored by metabolic cage for 150 min (n = 4). Asterisks (*) denote the level of statistical significance. *$p < 0.05$, **$p < 0.01$, ***$p < 0.001$. Data are presented as mean ± SEM. Statistical analyses were determined by unpaired two-tailed Student's *t*-test (**E**-**J**, **O**, **P**) and unpaired two-tailed Mann-Whitney test (**C**, bottom panel).

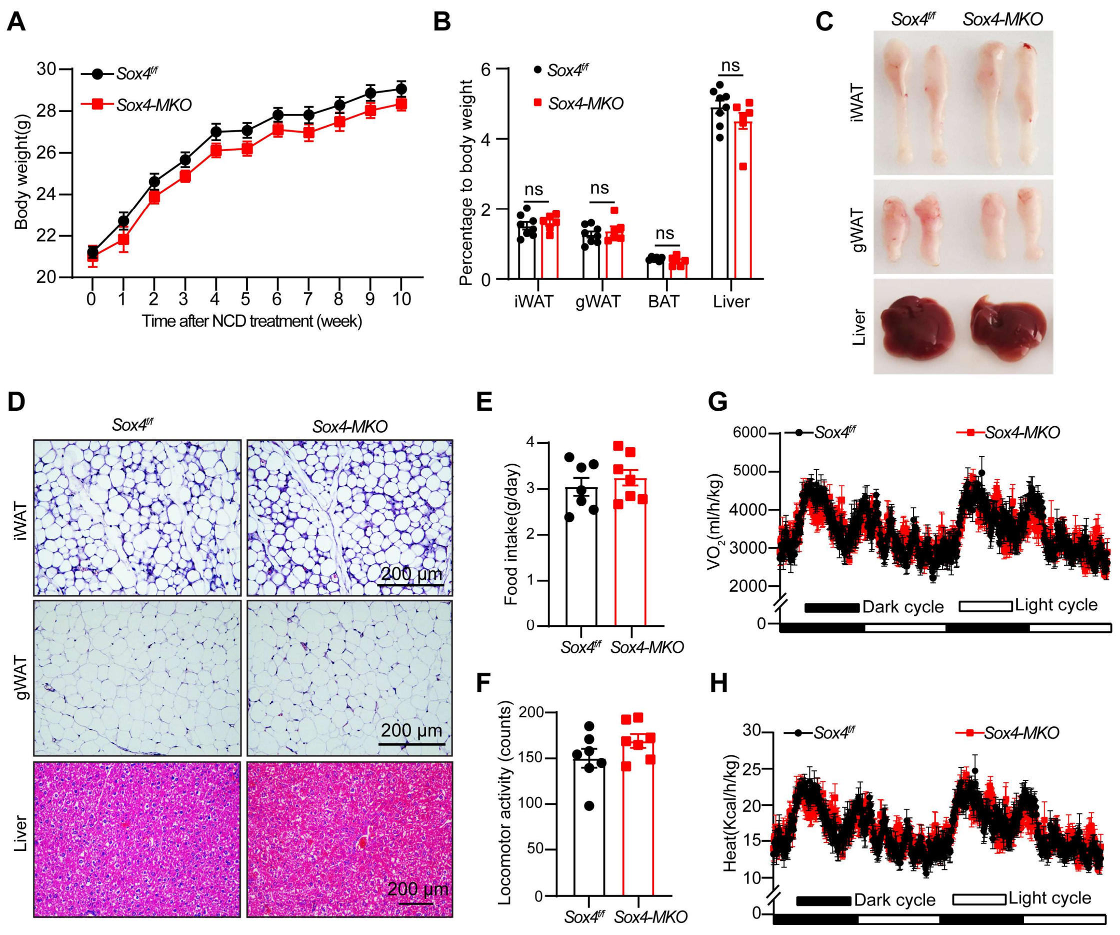

Fig.S4. ***Sox4-MKO* mice do not develop obesity on normal chow. (Related to Fig. 3)**

A The 6-week-old *Sox4$^{f/f}$ and Sox4-MKO* male mice (*Sox4$^{f/f}$*, n = 8; *Sox4-MKO*, n = 11) were subjected to chow feeding. Body weight was monitored every week. **B-D** The 10-week-old *Sox4-MKO* mice and their control male mice were sacrificed. The indicated ratios of tissue weight/body weight were shown in (**B**) (*Sox4$^{f/f}$*, n = 8; *Sox4-MKO*, n = 6). Representative images of iWAT, gWAT, and liver (**C**), and H&E staining (**D**) were shown. Scale bar, 200 μm. **E-H** The chow-fed mice were subjected to metabolic cage analysis. Food intake (**E**), locomotor activity (**F**), oxygen consumption (**G**) and heat production (**H**) of mice were measured in 2 consecutive days (n = 7). ns, no significance. Data are presented as mean ± SEM. Statistical analyses were determined by unpaired two-tailed Student's *t*-test (**A**, **B**, **E-H**).

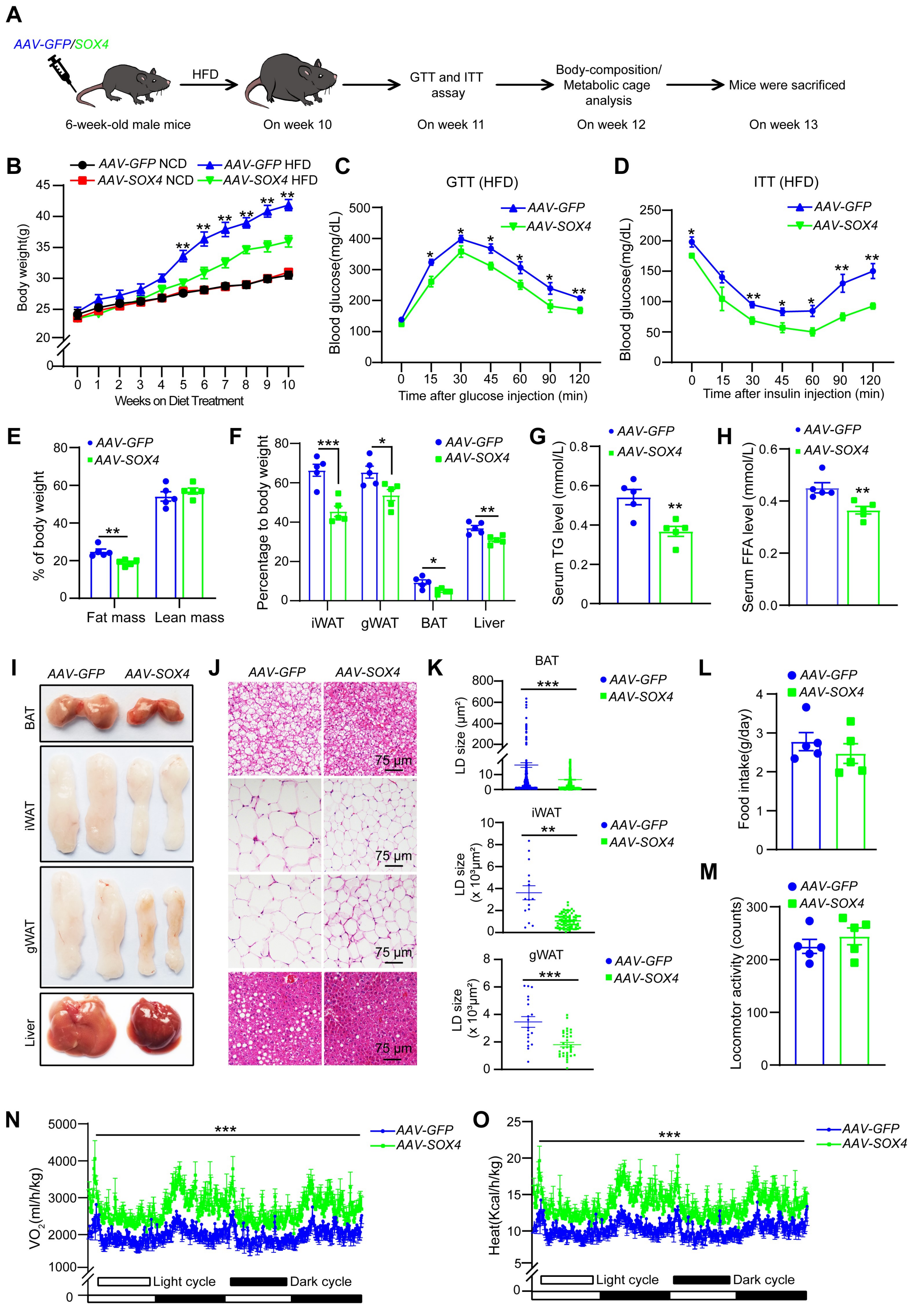

A
AAV-GFP/SOX4
HFD
6-week-old male mice
On week 10
GTT and ITT assay
On week 11
Body-composition/ Metabolic cage analysis
On week 12
Mice were sacrificed
On week 13
B
AAV-GFP NCD
AAV-SOX4 NCD
AAV-GFP HFD
AAV-SOX4 HFD
Body weight(g)
Weeks on Diet Treatment
C
GTT (HFD)
Blood glucose(mg/dL)
Time after glucose injection (min)
D
ITT (HFD)
Blood glucose(mg/dL)
Time after insulin injection (min)
E
% of body weight
Fat mass
Lean mass
F
Percentage to body weight
iWAT
gWAT
BAT
Liver
G
Serum TG level (mmol/L)
H
Serum FFA level (mmol/L)
I
BAT
iWAT
gWAT
Liver
J
75 μm
K
LD size (μm²)
LD size (x 10³μm²)
L
Food intake(g/day)
M
Locomotor activity (counts)
N
VO2(ml/h/kg)
Light cycle
Dark cycle
O
Heat(Kcal/h/kg)

Fig.S5. Overexpression of SOX4 in BAT attenuates HFD-induced obesity. (Related to Fig. 3)

The adeno-associated virus (AAV) system with *Ucp1* mini-promoter and enhancer was used to deliver GFP (*AAV-GFP*) or SOX4 (*AAV-SOX4*) into 6-week-old male mice via tail vein injection. After that, mice were fed with NCD or HFD. **A** Schematic figure showed *AAV-GFP* and *AAV-SOX4* mice were subjected to HFD feeding, and indicated experiment were performed at the indicated times. **B** The weight gain in chow or HFD feeding *AAV-SOX4* mice and control groups (n = 5). **C**, **D** GTT and ITT of mice after 11 weeks of HFD treatment (n = 5). **E** On week 12, the fat mass and the lean mass of *AAV-GFP* and *AAV-SOX4* mice were measured by Yuyan MRI analyzer (n=5). **F**-**K** At the end of the experiment, mice were euthanized on week 13, and blood and indicated tissues were collected. The mass of various fat pads and liver were normalized to body weight of mice (**F**) (n = 5). Serum levels of TG (**G**) and FFA (**H**) were measured (n = 5). Representative appearance (**I**) and H&E staining were shown in (**J**). The LDs sizes were quantified and shown in (K). Scale bar, 75 μm. **L-O** On week 12, the HFD-fed *AAV-GFP* and *AAV-SOX4* mice were subjected to metabolic cage analysis. Food intake (**L**), locomotor activity (**M**), oxygen consumption (**N**) and heat production (**O**) of mice were measured in 2 consecutive days (n = 5). Asterisks (*) denote the level of statistical significance. *$p < 0.05$, **$p < 0.01$, ***$p < 0.001$. Data are presented as mean ± SEM. Statistical analyses were determined by unpaired two-tailed Student's *t*-test (**B**-**G**, **K**, bottom panel; **L**-**O**) and unpaired two-tailed Mann-Whitney test (**H**; **K**, top panel and middle panel).

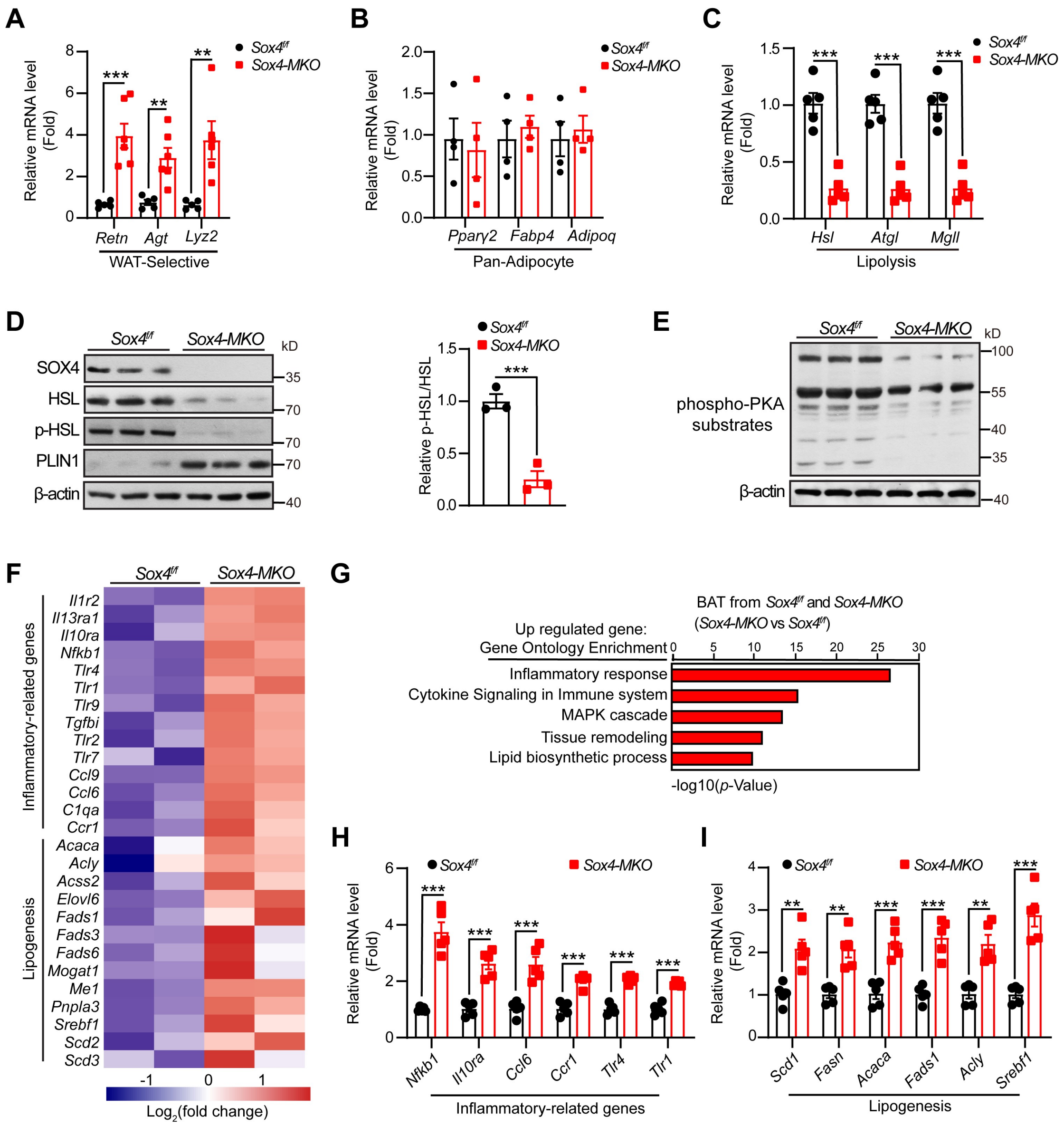
A
B
C
Relative mRNA level (Fold)
Sox4f/f
Sox4-MKO
Retn
Agt
Lyz2
WAT-Selective
Pparγ2
Fabp4
Adipoq
Pan-Adipocyte
Hsl
Atgl
Mgll
Lipolysis
D
SOX4
HSL
p-HSL
PLIN1
β-actin
kD
Relative p-HSL/HSL
E
phospho-PKA substrates
F
Inflammatory-related genes
Lipogenesis
Log2(fold change)
G
BAT from Sox4f/f and Sox4-MKO (Sox4-MKO vs Sox4f/f)
Up regulated gene: Gene Ontology Enrichment
Inflammatory response
Cytokine Signaling in Immune system
MAPK cascade
Tissue remodeling
Lipid biosynthetic process
-log10(p-Value)
H
Nfkb1
Il10ra
Ccl6
Ccr1
Tlr4
Tlr1
Inflammatory-related genes
I
Scd1
Fasn
Acaca
Fads1
Acly
Srebf1
Lipogenesis

Fig.S6. Loss of SOX4 downregulates lipolysis genes and upregulates lipogenesis genes. (Related to Fig. 4)

A-C qPCR analysis of WAT-selective genes in BAT of 9-week-old *Sox4-MKO* and control mice (*Sox4*$^{f/f}$, n = 5; *Sox4-MKO*, n = 6) (**A**). qPCR analysis of pan-adipocyte genes in BAT of 9-week-old *Sox4-MKO* and control mice (n = 4) (**B**). qPCR analysis of lipolysis gene in BAT of 9-week-old male *Sox4-MKO* and control littermates (n = 5) (**C**). *18S* was used as an invariant control. The mRNA levels in control mice were normalized to 1.0. **D** Western blot analysis of SOX4, HSL, p-HSL and PLIN1 protein in BAT from 9-week-old male *Sox4-MKO* and control mice (n = 3) (**D**, left). The levels of p-HSL relative to HSL were quantified using Image J (**D**, right). **E** The protein level of phospho-PKA substrates in BAT from 9-week-old male *Sox4-MKO* and control mice (n = 3). **F**, **G** Heat map analysis of the lipogenesis and inflammation genes (**F**). The up-regulated genes were used for clustering analysis and were plotted in (**G**). **H**, **I** qPCR analysis of inflammation genes (**H**) and lipogenesis genes (**I**) in BAT of 9-week-old male *Sox4-MKO* and control littermates (n = 5). *18S* was used as an invariant control. The mRNA levels in control mice were normalized to 1.0. Asterisks (*) denote the level of statistical significance. **$p < 0.01$, ***$p < 0.001$. Data are presented as mean ± SEM. Statistical analyses were determined by unpaired two-tailed Student's *t*-test (**A**-**C**, **D**, right; **H**, **I**).

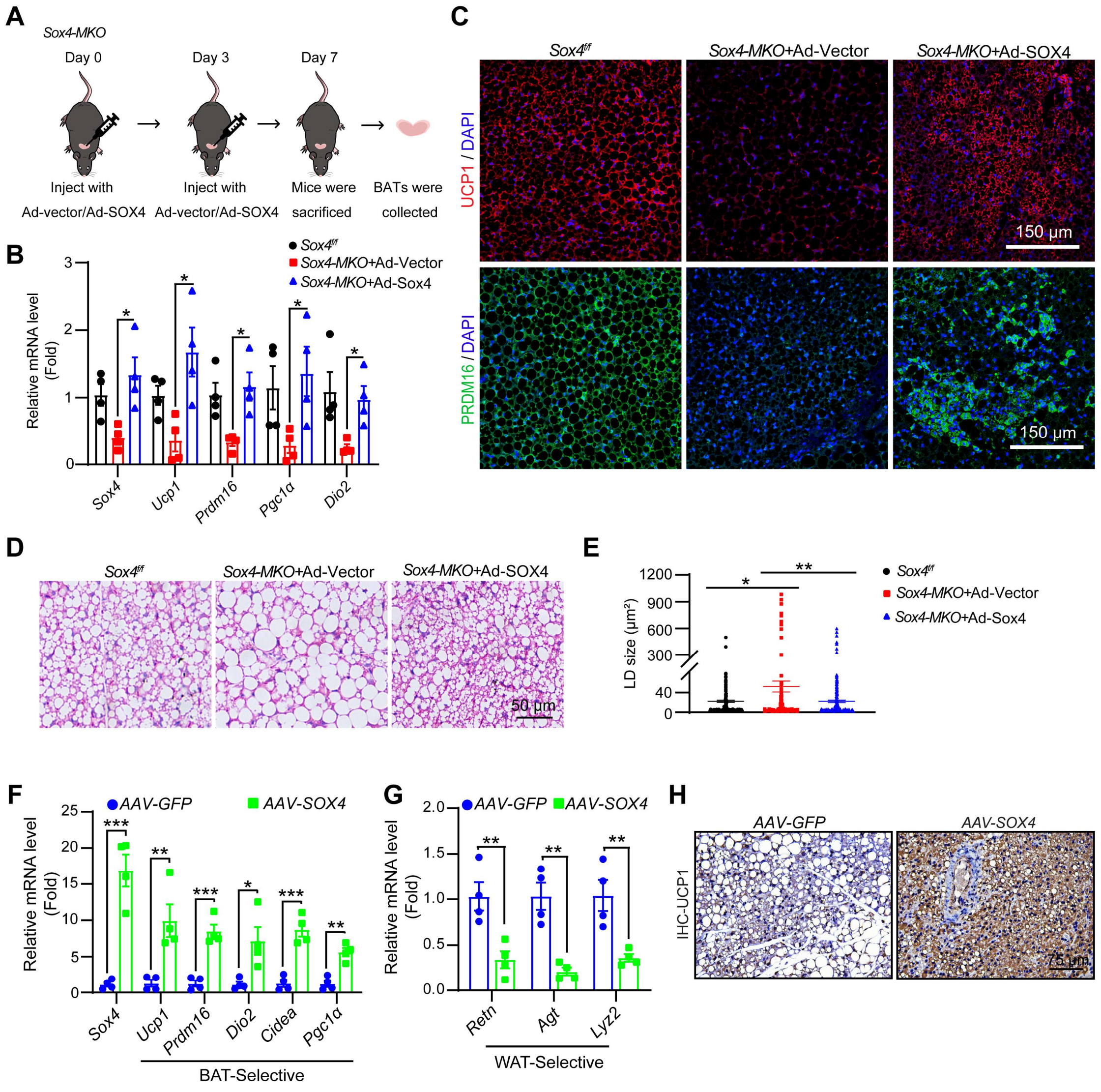
A
Sox4-MKO
Day 0
Day 3
Day 7
Inject with Ad-vector/Ad-SOX4
Inject with Ad-vector/Ad-SOX4
Mice were sacrificed
BATs were collected
B
Relative mRNA level (Fold)
Sox4f/f
Sox4-MKO+Ad-Vector
Sox4-MKO+Ad-Sox4
Sox4
Ucp1
Prdm16
Pgc1α
Dio2
C
Sox4f/f
Sox4-MKO+Ad-Vector
Sox4-MKO+Ad-SOX4
UCP1 / DAPI
PRDM16 / DAPI
150 μm
150 μm
D
Sox4f/f
Sox4-MKO+Ad-Vector
Sox4-MKO+Ad-SOX4
50 μm
E
LD size (μm²)
Sox4f/f
Sox4-MKO+Ad-Vector
Sox4-MKO+Ad-Sox4
F
AAV-GFP
AAV-SOX4
Relative mRNA level (Fold)
Sox4
Ucp1
Prdm16
Dio2
Cidea
Pgc1α
BAT-Selective
G
AAV-GFP
AAV-SOX4
Relative mRNA level (Fold)
Retn
Agt
Lyz2
WAT-Selective
H
AAV-GFP
AAV-SOX4
IHC-UCP1
75 μm

Fig.S7. SOX4 is required for BAT-selective thermogenic genes expression. (Related to Fig. 4)

A-E Adenovirus expressing vector or SOX4 were injected into BATs of 8-week-old *Sox4-MKO* male mice at Day 0 and Day 3. At day 7, mice were euthanized and BATs were collected (**A**). The mRNA levels of thermogenic genes were analyzed by qPCR (n = 4) (**B**). Immunofluorescence analysis for UCP1 (top) and PRDM16 (bottom) expression in BAT were shown in (**C**) (Scale bar, 150 μm). Representative H&E staining of BATs were shown in (**D**) (Scale bars, 50 μm). LDs sizes in BAT were shown in (**E**). *18S* was used as an invariant control. The mRNA levels in control mice were normalized to 1.0. **F**, **G** Total mRNA was extracted from BAT of *AAV-GFP* or *AAV-SOX4* mice described in method. The relative mRNA levels of BAT-selective genes (**F**) and WAT-selective (**G**) genes were analyzed by qPCR (n = 4). *18S* was used as an invariant control. The mRNA levels in control mice were normalized to 1.0. **H** Immunohistochemistry staining of UCP1 in BAT from *AAV-GFP* or *AAV-SOX4* mice. Scale bars, 75 μm. Asterisks (*) denote the level of statistical significance. *$p < 0.05$, **$p < 0.01$, ***$p < 0.001$. Data are presented as mean ± SEM. Statistical analyses were determined by unpaired two-tailed Student's *t*-test (**F**, **G**), one-way ANOVA followed by Tukey's test (**B**), and Kruskal-Wallis test with Dunn's multiple comparisons test (**E**).

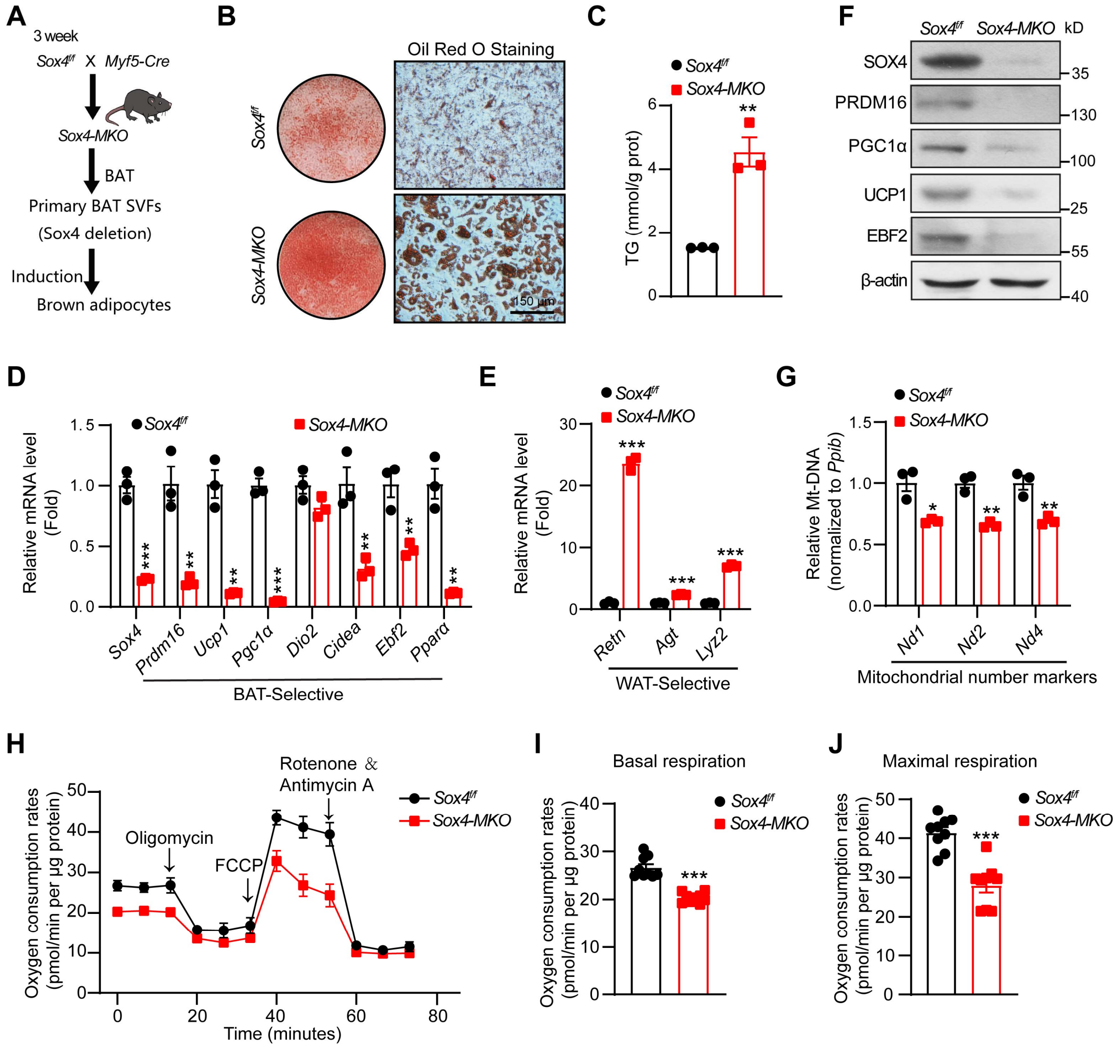
A
3 week
Sox4f/f X Myf5-Cre
Sox4-MKO
BAT
Primary BAT SVFs
(Sox4 deletion)
Induction
Brown adipocytes
B
Oil Red O Staining
Sox4f/f
Sox4-MKO
150 μm
C
Sox4f/f
Sox4-MKO
TG (mmol/g prot)
F
Sox4f/f
Sox4-MKO
kD
SOX4
PRDM16
PGC1α
UCP1
EBF2
β-actin
D
Relative mRNA level (Fold)
Sox4 Prdm16 Ucp1 Pgc1α Dio2 Cidea Ebf2 Pparα
BAT-Selective
E
Retn Agt Lyz2
WAT-Selective
G
Relative Mt-DNA (normalized to Ppib)
Nd1 Nd2 Nd4
Mitochondrial number markers
H
Oxygen consumption rates (pmol/min per μg protein)
Oligomycin
FCCP
Rotenone & Antimycin A
Time (minutes)
I
Basal respiration
J
Maximal respiration

Fig.S8. SOX4 is required for brown adipocytes differentiation *in vitro*. (Related to Fig. 5)

A-G The BAT SVF cells isolated from 3-week-old *Sox4-MKO* or control mice were induced to differentiate into mature brown adipocytes (**A**). On day 6 of differentiation, cells were harvested for Oil Red O staining (Scale bar, 150 μm) (**B**), and quantifications of triglyceride content were shown in (**C**) (n = 3). The mRNA levels of BAT-selective genes (**D**) and WAT-selective genes (**E**) were analyzed by qPCR (n = 3). *18S* was used as an invariant control. The mRNA levels in control cells were normalized to 1.0. The protein levels of PRDM16, PGC1α, EBF2 and UCP1 were analyzed by western blot (**F**). Mitochondrial-specific transcripts (*Nd1*, *2* and *4*) were measured by qPCR (**G**) (n = 3). *Ppib* was used as an invariant control. The mRNA levels in control cells were normalized to 1.0. **H**-**J** The OCR in differentiated primary brown adipocytes were shown in (**H**). The basal and maximal respiration rates in brown adipocytes were shown in (**I**) and (**J**), respectively (n = 3). Asterisks (*) denote the level of statistical significance. *$p < 0.05$, **$p < 0.01$, ***$p < 0.001$. Data are presented as mean ± SEM. Statistical analyses were determined by unpaired two-tailed Student's *t*-test (**C**-**E**, **G**, **I**, **J**).

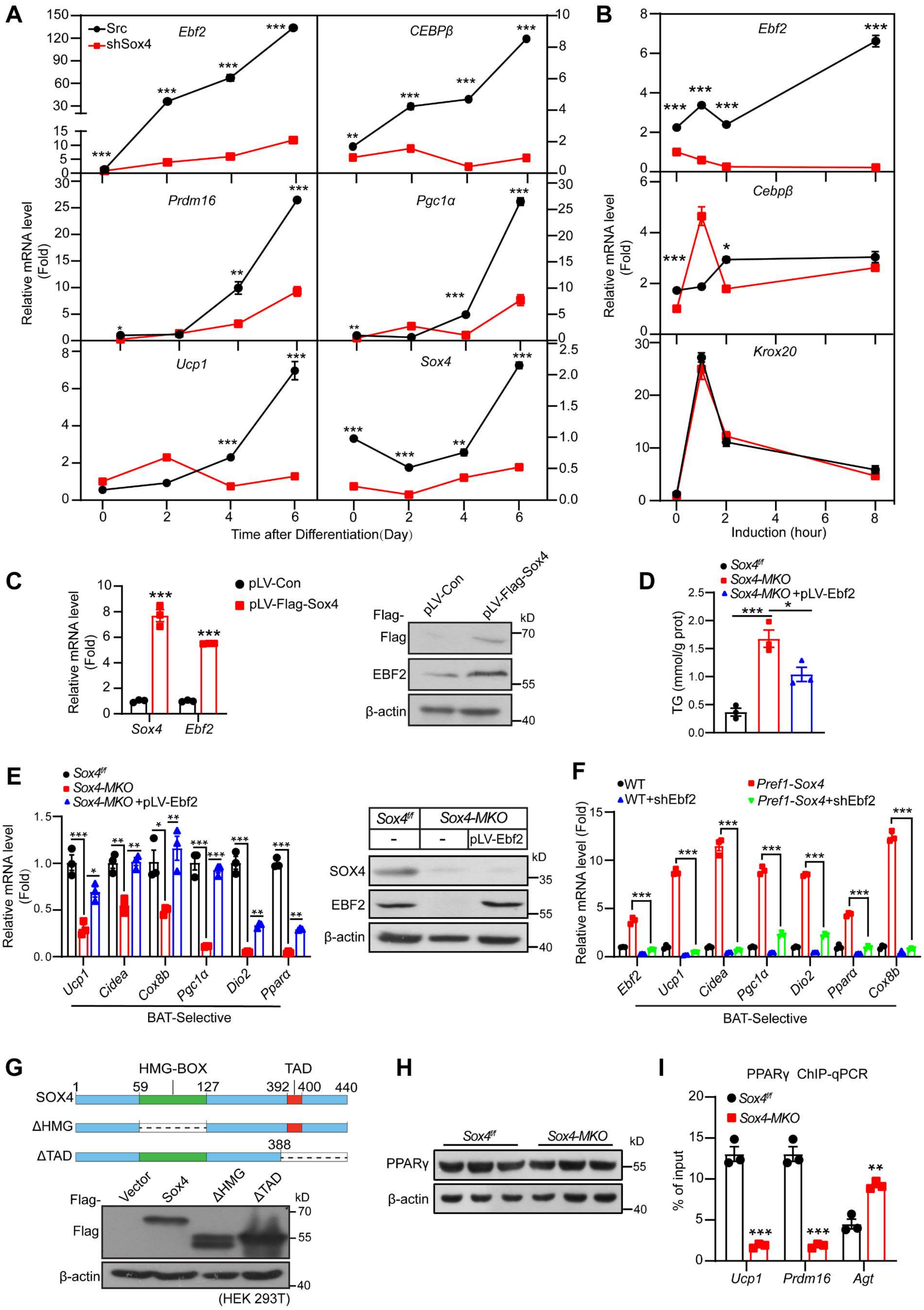

A
Src
shSox4
Ebf2
CEBPβ
Prdm16
Pgc1α
Ucp1
Sox4
Relative mRNA level (Fold)
Time after Differentiation(Day)
B
Ebf2
Cebpβ
Krox20
Relative mRNA level (Fold)
Induction (hour)
C
pLV-Con
pLV-Flag-Sox4
Relative mRNA level (Fold)
Sox4
Ebf2
Flag-
Flag
EBF2
β-actin
kD
D
Sox4f/f
Sox4-MKO
Sox4-MKO +pLV-Ebf2
TG (mmol/g prot)
E
Sox4f/f
Sox4-MKO
Sox4-MKO +pLV-Ebf2
Relative mRNA level (Fold)
Ucp1
Cidea
Cox8b
Pgc1α
Dio2
Pparα
BAT-Selective
pLV-Ebf2
SOX4
EBF2
β-actin
F
WT
WT+shEbf2
Pref1-Sox4
Pref1-Sox4+shEbf2
Relative mRNA level (Fold)
Ebf2
Ucp1
Cidea
Pgc1α
Dio2
Pparα
Cox8b
BAT-Selective
G
HMG-BOX
TAD
SOX4
ΔHMG
ΔTAD
Flag-
Vector
Sox4
ΔHMG
ΔTAD
Flag
β-actin
(HEK 293T)
H
Sox4f/f
Sox4-MKO
PPARγ
β-actin
kD
I
PPARγ ChIP-qPCR
Sox4f/f
Sox4-MKO
% of input
Ucp1
Prdm16
Agt

Fig.S9. SOX4 is required for transcription of EBF2 in brown adipocyte differentiation. (Related to Fig. 6)

A, **B** Control (scrambled) or Sox4-knockdown (shSox4) BAT SVF cells were set up and subjected to differentiation. Cells were collected on days 0, 2, 4 and 6 (**A**) or 0, 1, 2, 8 h (**B**) after differentiation and the indicated transcripts were analyzed by RT-qPCR (n = 3). *18S* was used as an invariant control. The mRNA levels in control cells were normalized to 1.0. **C** BAT SVF cells were infected with *Sox4* expressing lentivirus or control lentivirus. 48 h later, cells were harvested and the mRNA and protein levels of SOX4 and EBF2 were analyzed (n = 3). *18S* was used as an invariant control. The mRNA levels in control cells were normalized to 1.0. **D**, **E** On day 0, Control, *Sox4-MKO* and EBF2-overexpression *Sox4-MKO* BAT SVF cells were subjected into brown adipocyte differentiation. On day 6, cells were harvested for triglyceride content analysis (**D**) and qPCR analysis (**E**) (n = 3). *18S* was used as an invariant control. The mRNA levels in control cells were normalized to 1.0. **F** BAT SVF cells isolated from 3-week-old *Pref1-Sox4* mice and control mice were infected with scrambled or shEbf2 lentivirus and then differentiated for 6 days. Cells were harvested for qPCR analysis of BAT-selective genes (n = 3). *18S* was used as an invariant control. The mRNA levels in control cells were normalized to 1.0. **G** An illustration showed the structure of the mouse SOX4 protein and its truncated isoforms (ΔHMG and ΔTAD). ΔHMG indicated DNA binding domain deletion, and ΔTAD indicated transactivation domain deletion. Next, HEK293T cells were transfected with WT or mutant (ΔHMG and ΔTAD) plasmids. After 48 h, cells were harvested, and the protein levels were analyzed by western blot. **H** Protein levels of PPARγ in BAT of 9-week-old male *Sox4-MKO* and control

littermates (n = 3). **I** ChIP-qPCR analysis of PPARγ binding to the promoters of *Prdm16*, *Ucp1* and *Agt* in BATs from 9-week-old male *Sox4-MKO* and control littermates (n = 3). Asterisks (*) denote the level of statistical significance. *$p < 0.05$, **$p < 0.01$, ***$p < 0.001$. Data are presented as mean ± SEM. Statistical analyses were determined by unpaired two-tailed Student's *t*-test (**A**-**C**, **I**), one-way ANOVA followed by Tukey's test (**D**, **E**) and two-way ANOVA followed by Tukey's test (**F**).

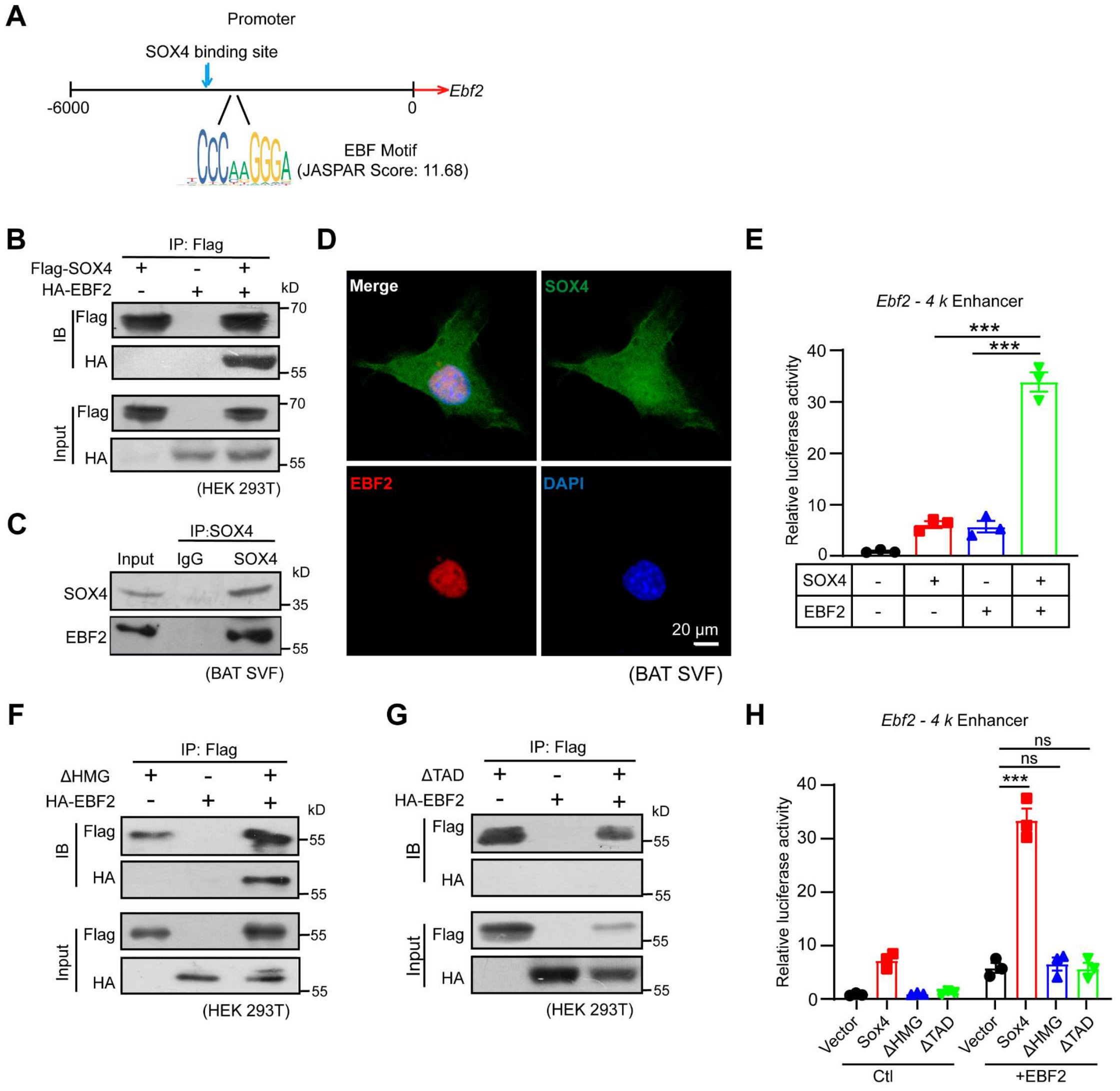
A
Promoter
SOX4 binding site
-6000
0
Ebf2
EBF Motif
(JASPAR Score: 11.68)
B
IP: Flag
Flag-SOX4 + - +
HA-EBF2 - + +
kD
IB
Flag
HA
Input
Flag
HA
70
55
70
55
(HEK 293T)
C
IP:SOX4
Input IgG SOX4
kD
SOX4
EBF2
35
55
(BAT SVF)
D
Merge
SOX4
EBF2
DAPI
20 μm
(BAT SVF)
E
Ebf2 - 4 k Enhancer
Relative luciferase activity
***
***
SOX4 - + - +
EBF2 - - + +
F
IP: Flag
ΔHMG + - +
HA-EBF2 - + +
kD
IB
Flag
HA
Input
Flag
HA
55
(HEK 293T)
G
IP: Flag
ΔTAD + - +
HA-EBF2 - + +
kD
IB
Flag
HA
Input
Flag
HA
55
(HEK 293T)
H
Ebf2 - 4 k Enhancer
Relative luciferase activity
ns
ns
***
Vector
Sox4
ΔHMG
ΔTAD
Ctl
+EBF2

Fig.S10. SOX4 cooperates with EBF2 to activate the transcription of Ebf2. (Related to Fig. 7)

A Schematic illustration of the adjacent binding sites of SOX4 and EBF2 at the region of Ebf2. **B** HEK293T cells were transfected with Flag-SOX4 and HA-EBF2. 48 h later, cells were harvested and subjected into immunoprecipitation (IP) with anti-flag beads. The input and pellet fractions were analyzed by western blot using indicated antibodies. **C** BAT SVF cells were lysed and subjected to immunoprecipitation using IgG or anti-SOX4 antibody. Input and pellet fractions were analyzed by western blot using indicated antibodies. **D** Immunofluorescence analysis showed the co-localization of SOX4 and EBF2 in the nucleus of BAT SVF cells. Scale bar, 20 μm. **E** Transcriptional activity of the -4kb region of *Ebf2* reporter in response to expression of SOX4, EBF2 or the combination of SOX4 and EBF2 in NIH3T3 cells (n = 3). **F**, **G** HEK293T cells were transfected with WT-Flag-ΔHMG (**F**) or ΔTAD (**G**) along with HA-EBF2. The cell lysates were subjected to IP with anti-Flag beads. The input and pellet fractions were analyzed by western blot using indicated antibodies. **H** Transcriptional activity of the -4kb region of *Ebf2* reporter in response to expression of SOX4, ΔHMG, ΔTAD, and EBF2 as indicated in NIH3T3 cells (n = 3). Asterisks (*) denote the level of statistical significance. ns, no significance; ***$p < 0.001$. Data are presented as mean ± SEM. Statistical analyses were determined by one-way ANOVA followed by Tukey's test (**E**, **H**).

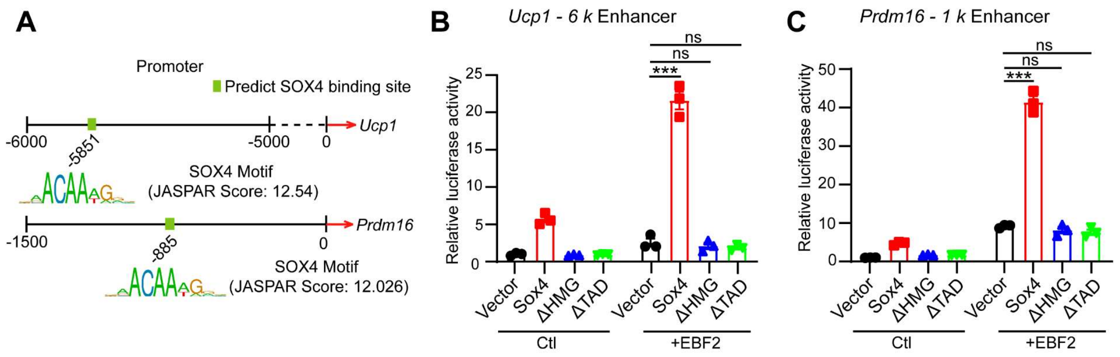

Fig.S11. SOX4 cooperates with EBF2 to promote transcription of thermogenic genes. (Related to Fig. 8)

A A schematic illustration showed that conservative binding sites of SOX4 in the *UCP1* and *Prdm16* promoter regions predicted by JASPAR. **B**, **C** Transcriptional activity of the -6kb region of *Ucp1* reporter (**B**) and -1kb region of *Prdm16* reporter (**C**) in NIH3T3 cells in response to expression of SOX4, ΔHMG or ΔTAD and EBF2 (n = 3). Asterisks (*) denote the level of statistical significance. ns, no significance; ***$p < 0.001$. Data are presented as mean ± SEM. Statistical analyses were determined one-way ANOVA followed by Tukey's test (**B**, **C**).
