# Supplementary material for: SOX4 facilitates brown fat development and maintenance through EBF2-mediated thermogenic gene program in mice
Source: Cell Death Differ. 2024 Oct 15;32(3):447–65. doi: 10.1038/s41418-024-01397-0 (PMC11893884; doi:10.1038/s41418-024-01397-0)

Supplementary original blots

Fig. 1B

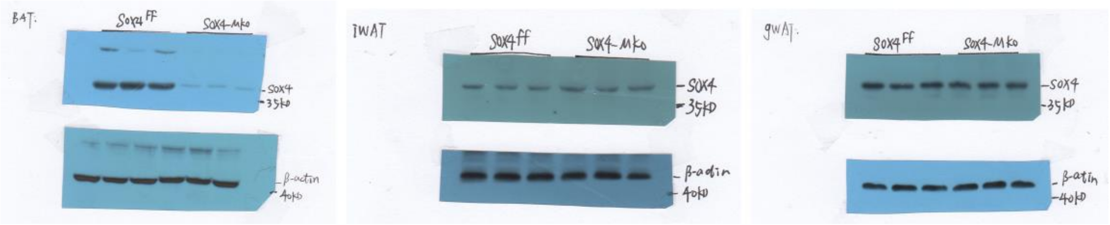

Fig. 1G, 1J and 1M

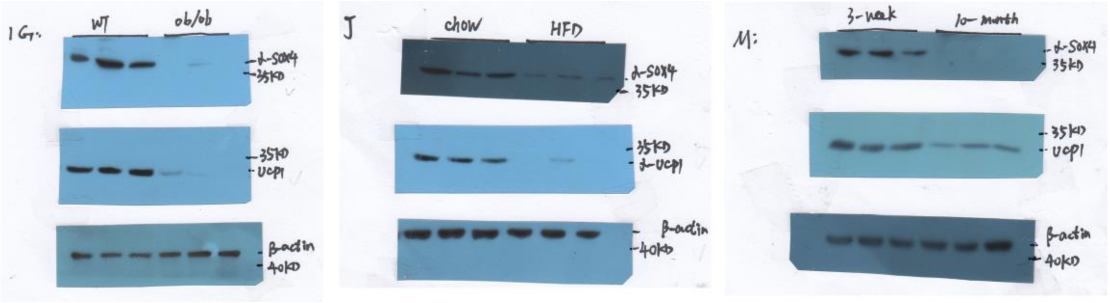

Fig. 4H, 4I

Fig. 4H

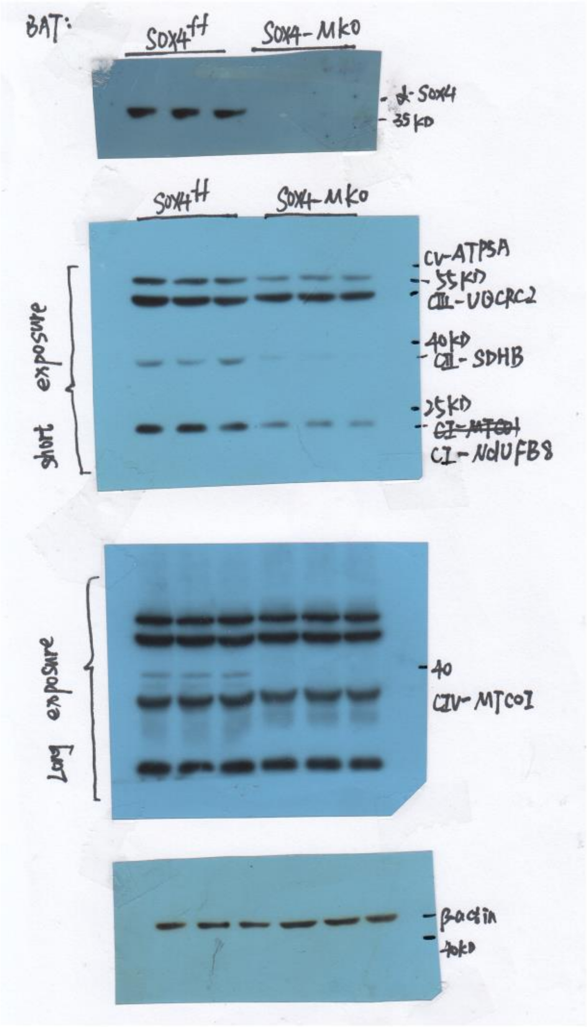

Fig. 4I

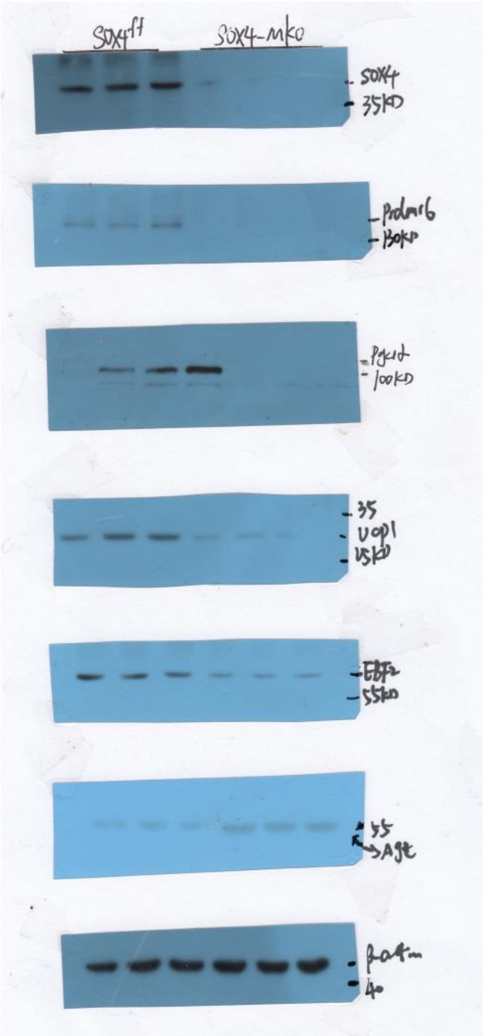

Fig. 5F

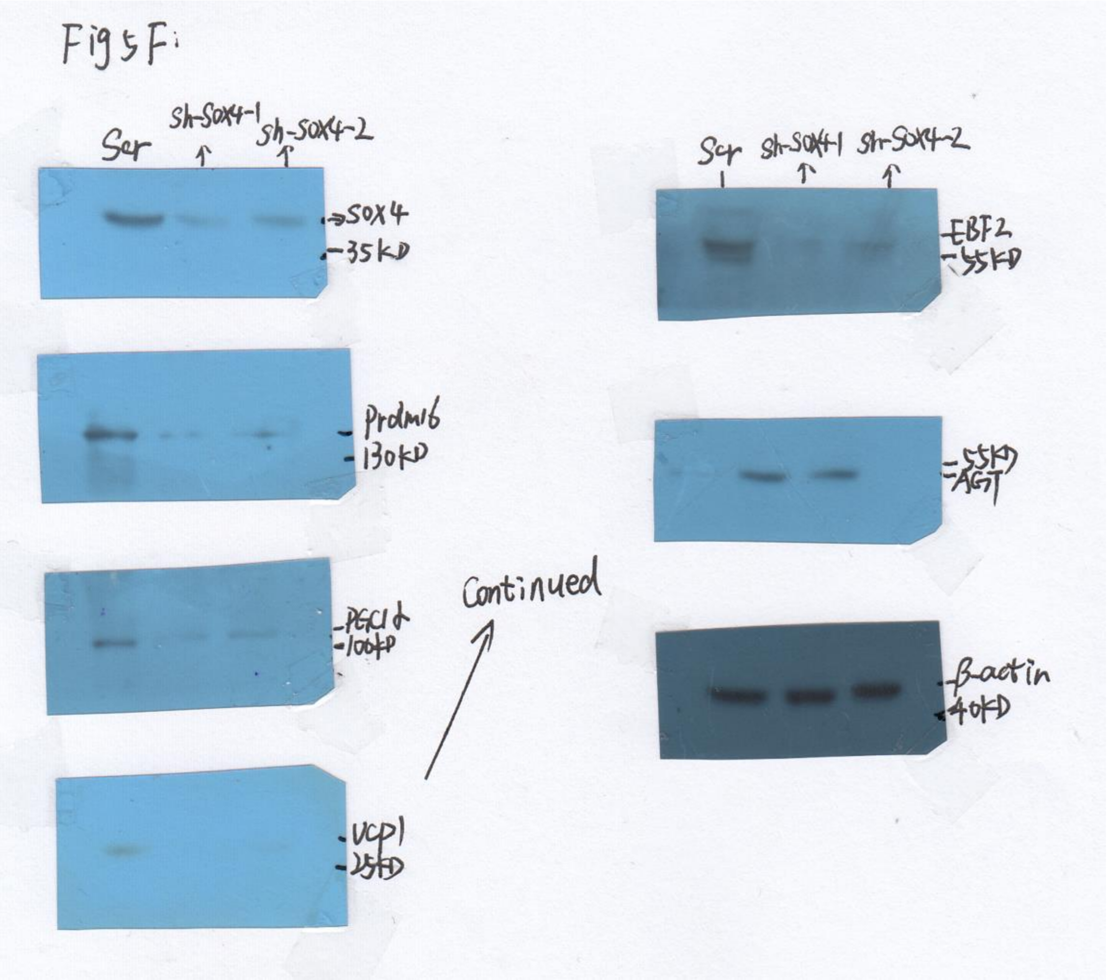
Fig 5F:
Scr
sh-SOX4-1
sh-SOX4-2
SOX4
35KD
Prdm16
130KD
PGC1α
100KD
UCP1
25KD
Continued
EBF2
55KD
55KD
AGT
β-actin
40KD

Fig. 7I

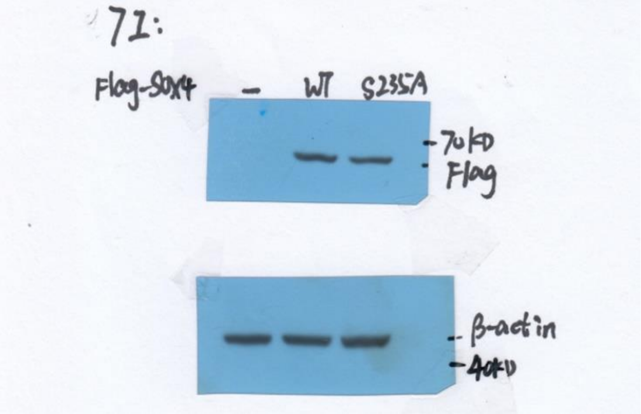
7I:
Flag-Sox4
–
WT
S235A
70KD
Flag
β-actin
40KD

Fig. 8B

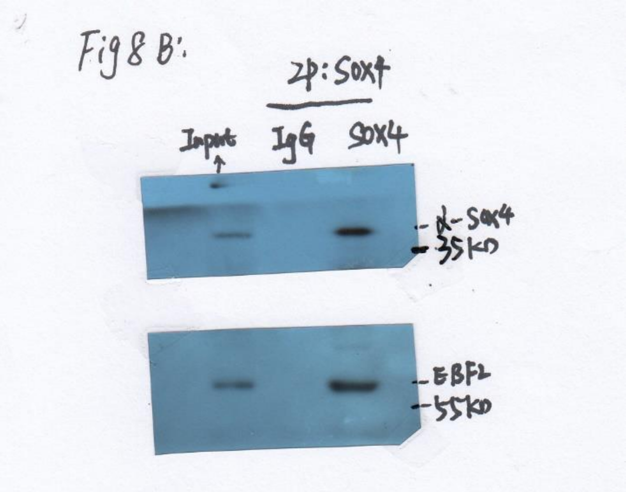

Fig. S1B, E

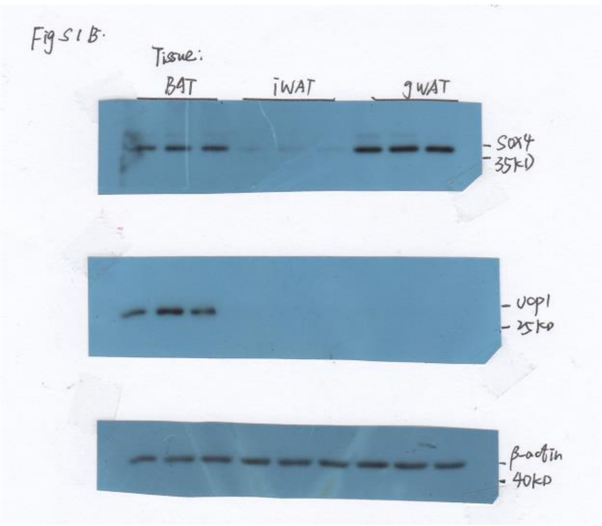
Fig S1B.
Tissue:
BAT
iWAT
gWAT
Sox4
35KD
Ucp1
25KD
β-actin
40KD

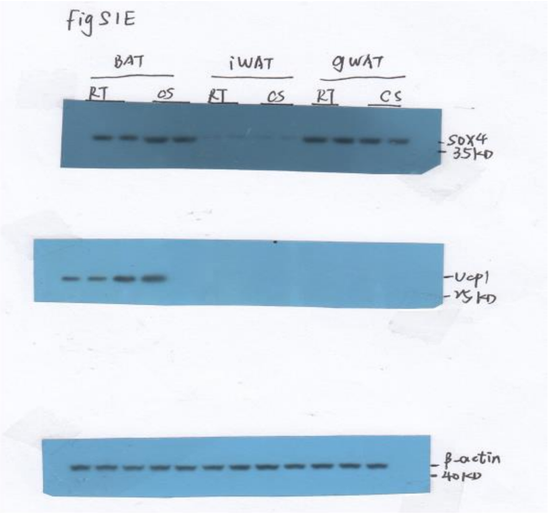
Fig S1E
BAT
iWAT
gWAT
RT
CS
RT
CS
RT
CS
Sox4
35KD
Ucp1
25KD
β-actin
40KD

Fig. S1J

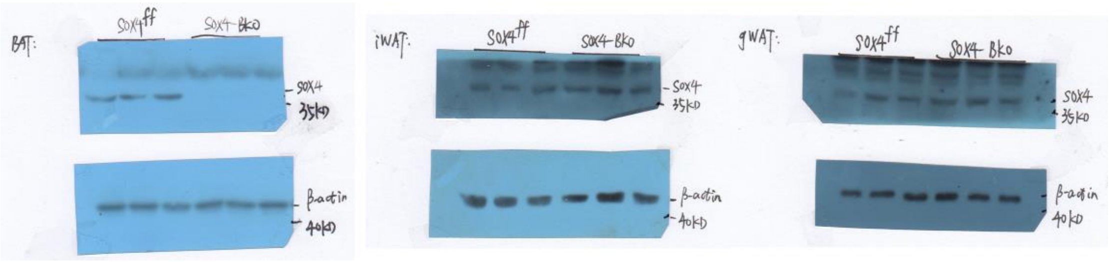

Fig. S3A, S3L

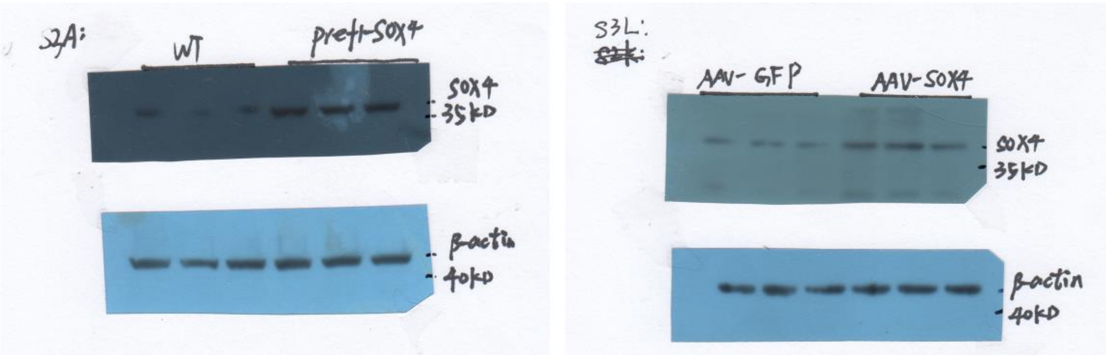

Fig. S6D, S6E

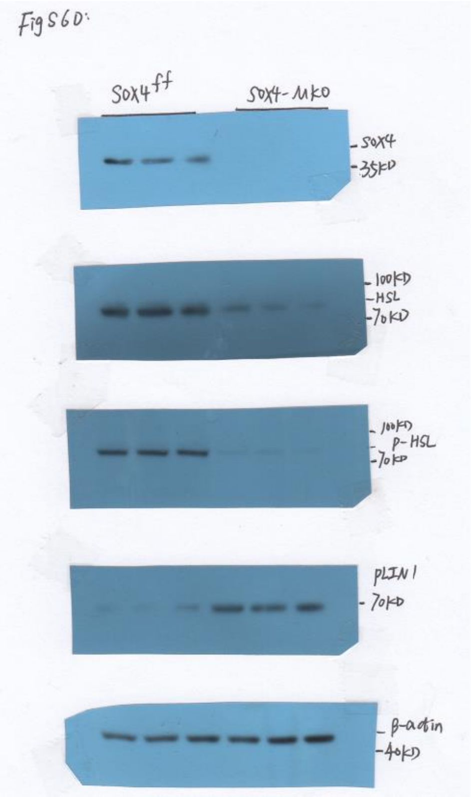
FigS6D:
Sox4ff
Sox4-MKO
Sox4
35KD
100KD
HSL
70KD
100KD
p-HSL
70KD
PLIN1
70KD
β-actin
40KD

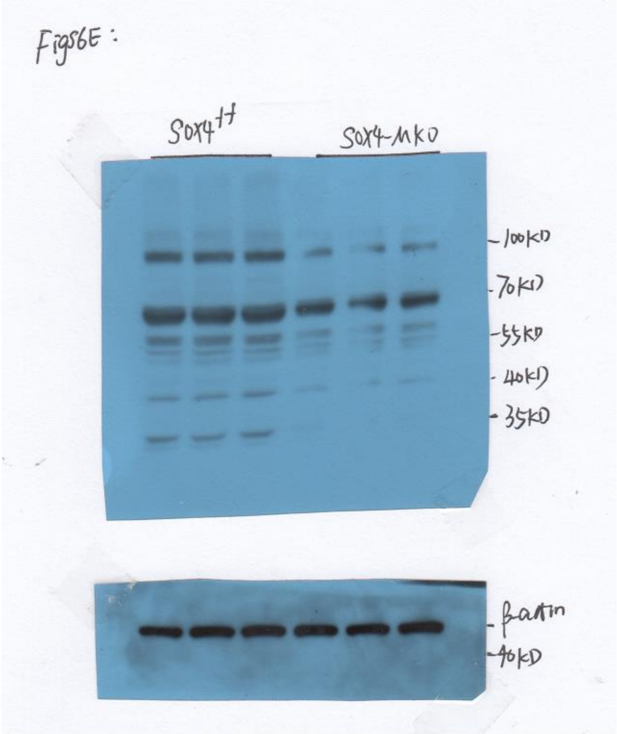
FigS6E:
Sox4ff
Sox4-MKO
100KD
70KD
55KD
40KD
35KD
β-actin
40KD

Fig. S8F

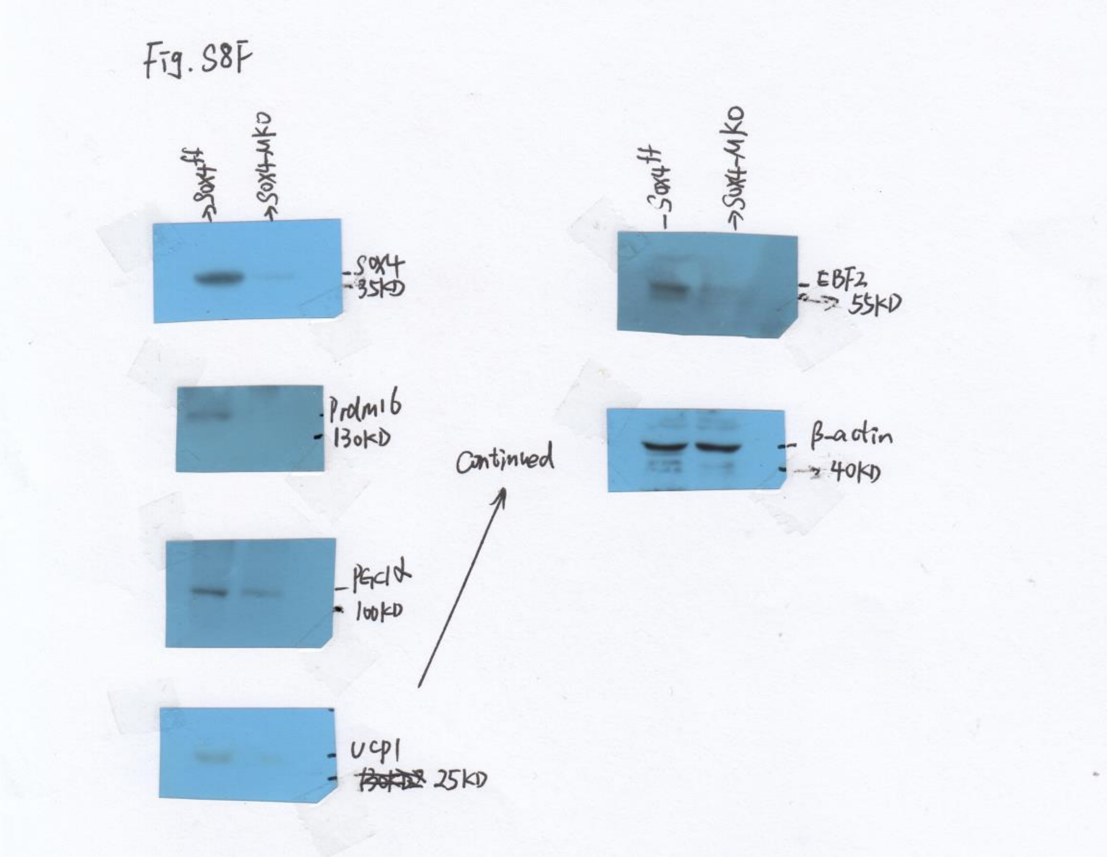
Fig. S8F
Sox4 f/f
Sox4-MKO
Sox4
35KD
Prdm16
130KD
PGC1α
100KD
UCP1
25KD
Continued
EBF2
55KD
β-actin
40KD

Fig. S9C, E, G and H

Fig. S9C

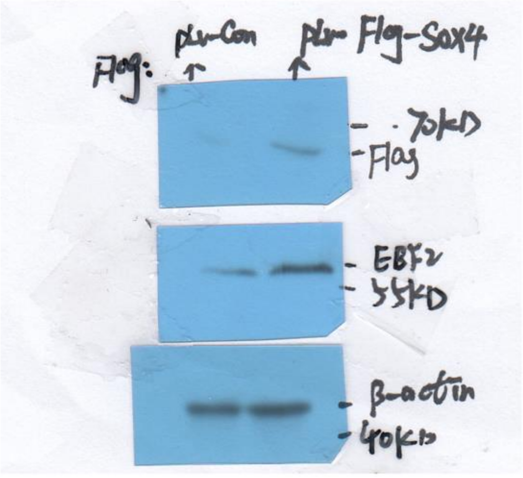

Fig. S9E

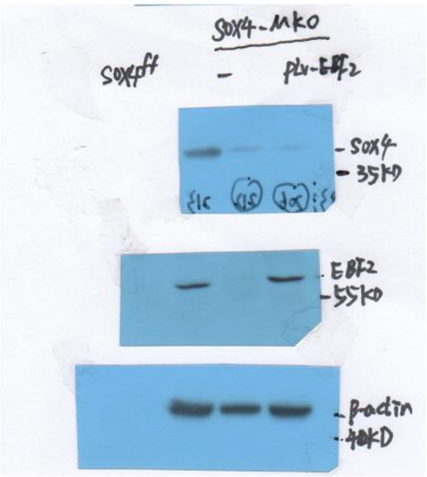

Fig. S9G

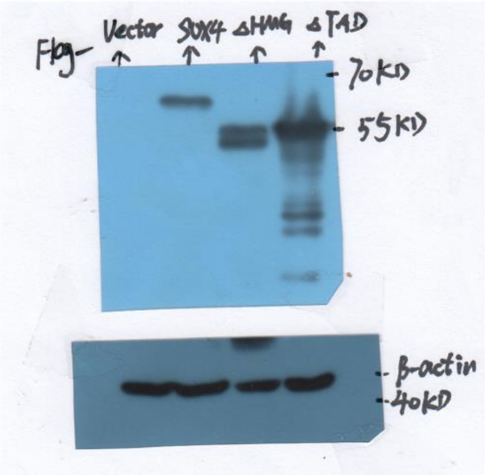

Fig. S9H

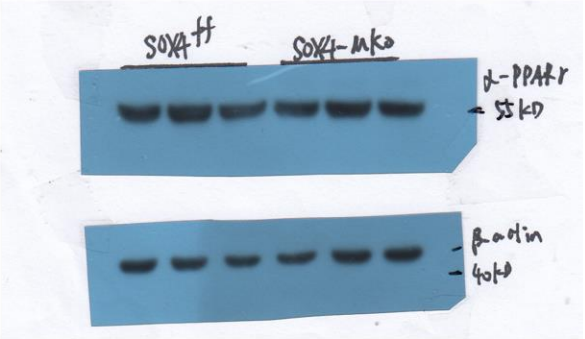

Fig. S10B, C, F and G

Fig. S10 B

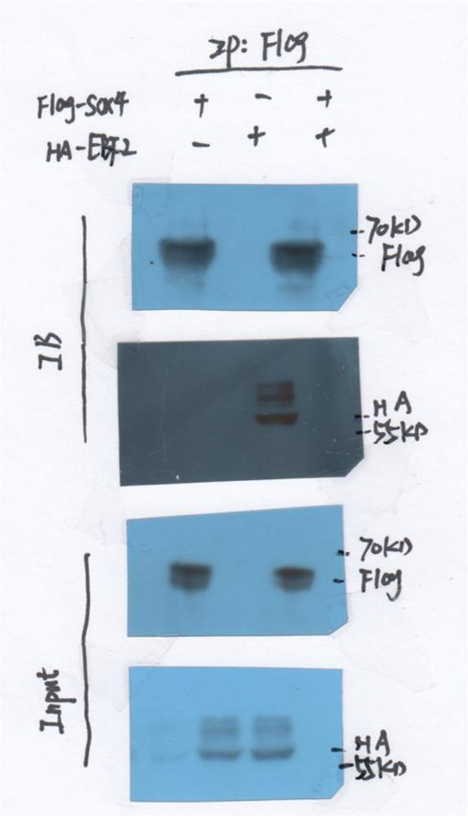

Fig. S10 C

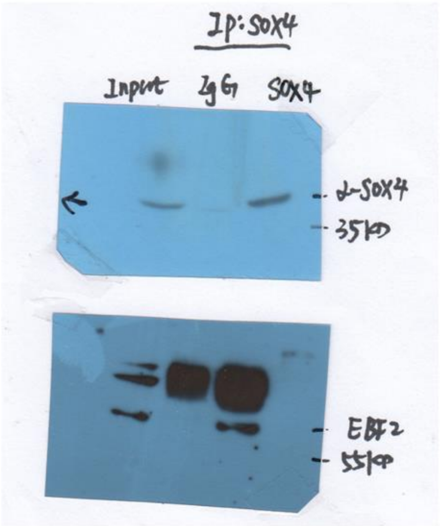

Fig. S10 F

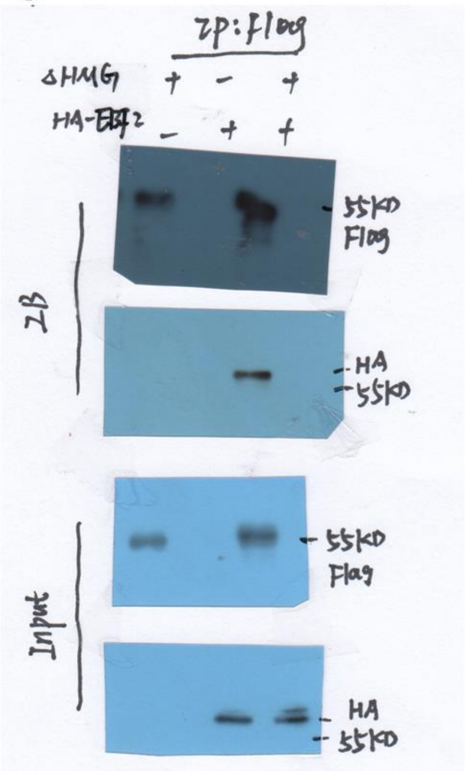

Fig. S10G

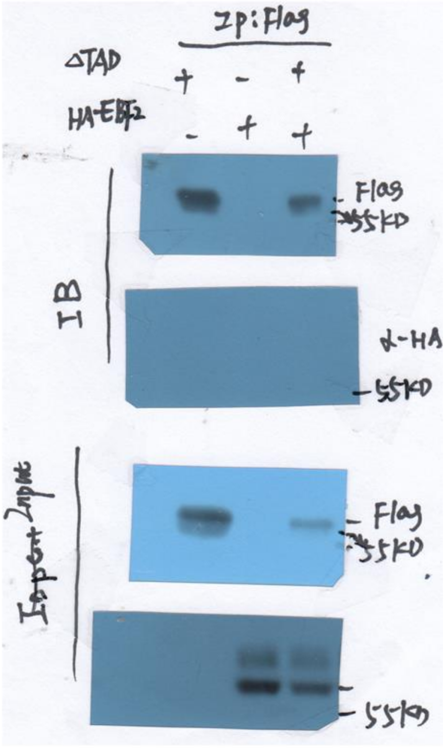

Supplement: Supplementary file 3 — Uncropped western blots [file 41418_2024_1397_MOESM3_ESM.pdf]
